# Pathology of African Swine Fever in Wild Boar Carcasses Naturally Infected with German Virus Variants

**DOI:** 10.3390/pathogens11111386

**Published:** 2022-11-20

**Authors:** Julia Sehl-Ewert, Paul Deutschmann, Angele Breithaupt, Sandra Blome

**Affiliations:** 1 Department of Experimental Animal Facilities and Biorisk Management, Friedrich-Loeffler-Institut, Südufer 10, 17493 Greifswald-Insel Riems, Germany; 2 Institute of Diagnostic Virology, Friedrich-Loeffler-Institut, Südufer 10, 17493 Greifswald-Insel Riems, Germany

**Keywords:** ASFV, pathology, Germany, virus variant, wild boar, natural infection

## Abstract

In 2020, African swine fever (ASF) was first identified in German wild boar, reaching a case number of about 4400 to date. Upon experimental infection, pathology is well documented; however, data on field infections are scarce in domestic pigs and not available from wild boar, respectively. Although the ASF viral genome is considered exceptionally stable, a total of five lineages with 10 distinct virus variants of genotype II have emerged in Eastern Germany. To investigate the pathology in naturally infected wild boar and to evaluate virus variants II, III and IV for their virulence, wild boar carcasses were obtained from three different outbreak areas. The carcasses underwent virological and pathomorphological investigation. The animals revealed characteristic ASF lesions of the highest severity accompanied by bacterial infections in several cases. In particular, wild boar infected with variant IV from Spree-Neiße (SN) district showed lower viral genome loads and total viral antigen scores, but simultaneously tended to reveal more chronic lesions. Our findings indicate a protracted course of the disease at least after infection with variant IV, but need confirmation under standardized experimental conditions. There is a strong need to monitor differences in the virulence among variants to identify potential attenuation that might complicate diagnosis. In addition, veterinarians, hunters and farmers need to be made aware of less acute courses of ASF to consider this as an important differential to chronic classical swine fever.

## 1. Introduction

Since its first occurrence in Georgia in 2007, African swine fever (ASF) has continuously spread from the Trans-Caucasian region to Russia, and, in 2014, further to countries of Europe [1]. In September 2020, the disease was confirmed for the first time in a wild boar found in the Spree-Neiße (SN) district in Eastern Germany close to the German–Polish border [2]. To date, more than 4400 cases in German wild boar in the Eastern federal states Brandenburg, Saxony and Mecklenburg-Western Pomerania as well as seven outbreaks in domestic pig holdings located in Brandenburg, Mecklenburg-Western Pomerania, Baden-Wurttemberg and Lower Saxony have been officially identified (https://tsis.fli.de/Reports/Info.aspx, accessed on 9 September 2022).

African swine fever, which is caused by the large, enveloped, double-stranded DNA African swine fever virus (ASFV), can occur as acute, subacute, chronic and subclinical disease courses depending on the virulence of the virus strain as well as on the age and immunological background of the animals [3]. In European countries except Sardinia, highly virulent virus strains of genotype II are prevalent in domestic and wild pigs typically causing acute-lethal disease similar to a hemorrhagic fever [4,5,6]. Genotype II strains were also identified in the German federal states of Brandenburg and Saxony including the outbreak areas Märkisch-Oderland (MOL), Oder-Spree (LOS), Spree-Neiße (SN) and Görlitz, in which, surprisingly, five lineages (I-V) including a total of ten viral variants (I, II, II.1, III, III.1, IV, IV.1, IV.2, IV.3, V) have emerged due to single nucleotide variations, insertions and deletions affecting different genes including five multigene families [7]. More specifically, variants III and IV comprise genetic variations in four multigene family (MGF) genes MGF360-10L, MGF360-15R, MGF100-3L and MGF505-4R while variant II shows variation only in the A240L gene coding for the ASFV thymidylate kinase. Whereas the functions of these genes are largely unknown, ASFV MGF360 and MGF505 have been associated with the virulence and pathogenicity of the virus [8,9]. Geographic mapping showed that variant II was predominantly spread in the outbreak area LOS, variant III in MOL and variant IV in the southern part of the outbreak area SN as well as in the federal state of Saxony.

To date, macroscopic pathological records of varying depths of detail largely exist only for experimentally ASFV-infected domestic pigs [10,11,12,13,14,15,16] and less frequently for wild boar [4,17,18,19,20], which is mainly due to the limited access to wild boar and the associated difficulties to keep them under experimental conditions. Moreover, histopathological data obtained from animal experiments are much less available, but gained importance in the last few years [16,18,21]. Very recently, the first three reports were published concerning naturally ASFV-infected domestic pigs from an outbreak in Vietnam, reporting on the clinical and pathological findings of succumbing and surviving pigs [21,22] and describing ASF-associated age-related lesions [23]. In contrast, descriptions of pathological findings of wild boar that succumb to infection under field conditions are completely missing although this animal species is of great relevance in the maintenance and spread of ASFV in Europe. Hence, the diversity and dimensions of ASFV-associated lesions in the field are only very sparsely represented urging more thorough investigations.

Based on this, we aimed to perform pathological examination of wild boar carcasses infected with ASFV to gain more profound knowledge of the pathology of the animals succumbing to ASF under natural conditions. We took the opportunity to analyze whether three different variations of the emerging virus variants in Germany may have an impact on the virulence of ASFV and the severity and duration of the disease. For this purpose, detailed pathological and molecular virological investigations were performed on wild boar carcasses infected with variants II, III and IV found in LOS, MOL and SN, respectively.

## 2. Material and Methods

### 2.1. Study Design

In accordance with the Animal Disease Crisis Unit of the federal states of Brandenburg and Saxony, sixteen wild boar carcasses were obtained from different outbreak areas (n = 7 from Landkreis Oder-Spree (LOS), n = 5 from Märkisch-Oderland (MOL), n = 4 from Spree-Neiße (SN)) between February and March 2021 where ASF virus variants II, III and IV have emerged as published previously [7]. Following legal requirements, the carcasses were tested positive for ASFV by the federal state laboratories of Brandenburg and Saxony. ASF diagnosis was confirmed by the national reference laboratory for ASF. The carcasses were transported to the Friedrich-Loeffler-Institut in compliance with national animal disease and hygiene regulations. The wild boar carcasses were examined in pathological and virological detail. Bacteriologic investigations of secondary bacterial infections were not performed for biosecurity reasons. Details on the cadaver material including location of origin, detection of virus variant, age, sex, weight and preservation status are given in Table 1.

#### 2.1.1. Pathological Examination

##### Necropsy

Full necropsies were performed on the wild boar carcasses (n = 16). The organ lesions were scored from 0 to 3 (0 = normal, 1 = mild, 2 = moderate, 3 = severe; unless not otherwise stated) as recently published [25] with the additional modifications shown in Table 2. Tissues samples including the popliteal lymph node, spleen, lung, kidney, liver, heart, brain (cerebellum and cerebrum) and adrenal gland were taken from wild boar and fixed in 10% neutral-buffered formalin for at least 3 weeks.

##### Histopathology and Immunohistochemistry

The tissue samples were embedded in paraffin wax and cut at 2–3 µm slices. Hematoxylin-eosin (HE) staining was performed to examine the main macroscopic lesions in more histological detail. To visualize viral antigens, anti-ASFV p72 immunohistochemistry was conducted on the respective organs as described earlier [17,18]. In brief, sections were treated with an in-house rabbit polyclonal primary antibody against the major capsid protein p72 of ASFV (diluted in TBS 1:1600, 1 h), followed by incubation with a secondary, biotinylated goat anti-rabbit IgG (Vector Laboratories, Burlingame, CA, USA; diluted in TBS in 1:200, 30 min). Positive antigen detection was visualized by the Avidin–Biotin Complex (ABC) method providing horseradish peroxidase that converted the added chromogen 3-amino-9-ethylcarbazole (AEC) into insoluble red-colored deposits at the reaction site. As negative control, consecutive sections were labeled with an irrelevant antibody (M protein of Influenza A virus, ATCC clone Hb64). An ASF positive control slide was included in each run.

##### Histopathology including Semiquantitative Antigen Scoring

The slides were scanned using a Hamamatsu S60 scanner and evaluated using NDPview.2 plus software (Version 2.8.24, Hamamatsu Photonics, K.K. Japan). While the histopathological lesions obtained on HE-stained sections were described only qualitatively (present/absent) due to autolysis-related limited assessability, the viral antigen content in the respective organ was determined on a semiquantitative scoring scale as previously published [18]. The most affected area (420 × 260 µm) per sample sections was scored with score 0 (no antigen), score 1 (1–3 positive cells), score 2 (4–15 cells) or score 3 (>16 cells). Cells with fine granular cytoplasmic labeling were considered positive whereas chromogen aggregations without cellular association were not counted.

#### 2.1.2. Detection of ASFV Genome

To determine the viral genome load, the tissue samples were homogenized in 1 mL of phosphate buffered saline with a metal bead using a TissueLyzer II (Qiagen GmbH, Hilden, Germany). Viral nucleic acids were extracted from blood and homogenized spleen, lung, liver, kidney, popliteal lymph node and brain with the NucleoMag Vet Kit (Machery-Nagel, Düren, Germany) on the KingFisher extraction platform (Thermo Scientific, Waltham, MA, USA). Quantitative real-time PCR (qPCR) was conducted according to the protocol published by King et al. [26] with an in-house full virus standard for determination of genome loads on a C1000 thermal cycler with the CFX96 Real-Time System (Biorad, Hercules, CA, USA).

#### 2.1.3. Detection of Anti-ASFV Antibodies

For investigation of ASFV-specific antibodies, an accredited indirect immunoperoxidase test (IPT) was applied according to the standard protocol SOP/CISA/ASF/IPT/1 provided by the European Reference laboratory for ASF with modifications regarding cell and virus type (https://asf-referencelab.info/asf/images/ficherosasf/PROTOCOLOS-EN/2021_UPDATE/SOP-ASF-IPT-1_2021.pdf, accessed on 4 April 2022). As sample material, plasma was obtained from EDTA blood by centrifugation at 18.000 g-force for 10 min from German wild boar carcasses and domestic pigs infected with ASFV “Estonia 2014” from a previous trial for comparison. Titers were determined semiquantitatively by endpoint dilution from 1:40 to 1:12,800.

#### 2.1.4. Statistical Analysis

Using GraphPad Prism (Version 8.4.2), statistical analysis was conducted to determine overall group differences in terms of viral genome load, viral antigen amount, macroscopic lesion scores and antibody titers. For this purpose, the non-parametric Kruskal–Wallis test with post hoc Dunn’s test was performed. A *p* value ≤ 0.05 was considered significant.

## 3. Results

### 3.1. Pathogen Detection in Blood and Tissues

**Figure 1:**
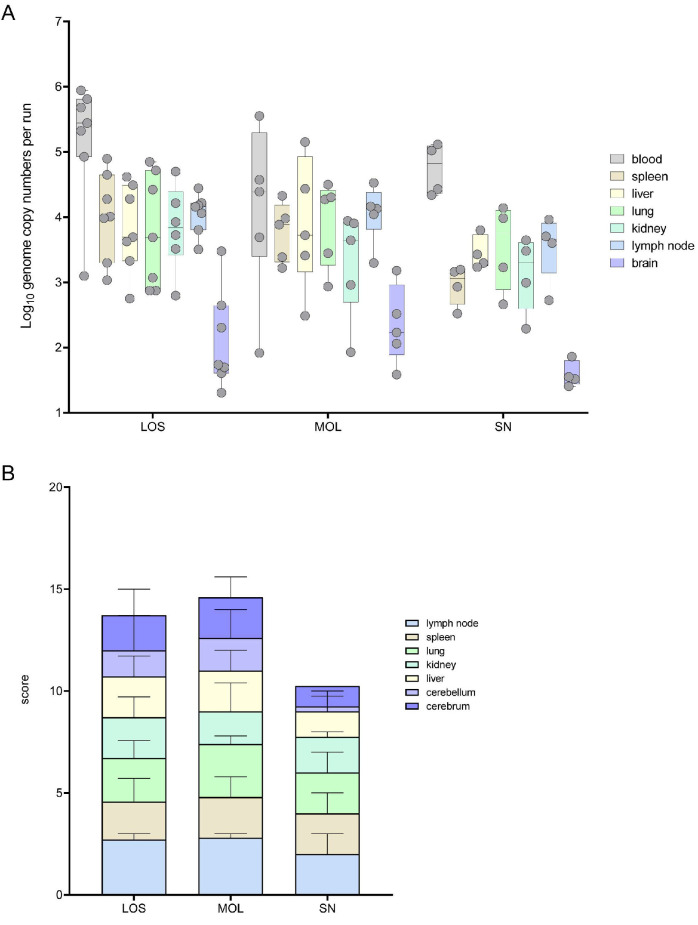
Pathogen detection in blood and tissue samples of ASFV-infected wild boar carcasses from LOS, MOL and SN. (**A**) Box plot presenting the individual viral genome load in blood and organ samples. (**B**) Corresponding stacked bar diagram showing the median viral antigen score with range per organ. Organs were scored on a scale from 0 to 3 based on the number of positively labeled cells in the most-affected tissue area per high power field.

Full necropsies were performed on all wild boar obtained from the outbreak areas LOS, MOL and SN to determine the amount of viral genome and antigen. The results are shown in Figure 1 and details are given in Supplementary Tables S1 and S2.

Viral genome could be found in all the samples of the infected wild boar. In general, the highest viral genome loads were detected in blood samples, varying between 1 × 10^2^ and 9 × 10^5^ genome copies (gc)/5 μL nucleic acid. In general, genome loads in most organ samples were roughly one logarithmic step lower than the corresponding blood samples. A lower mean viral genome load was detected in wild boar found in SN when compared to animals from LOS and MOL (Figure 1A).

The viral antigen score of selected tissue sections reflected the results obtained by qPCR. Consistent with the lower number of viral genome copies, wild boar from SN also reached lower viral antigen scores (Figure 1B). Details on immunohistochemistry are included in the histopathological evaluation of organ systems in the following section.

### 3.2. Pathological Assessment of Organ Systems

All carcasses were scored macroscopically based on a standardized scoring system [25] with further modifications as indicated in Table 2. Histopathological alterations were reported only as present/absent due to the reduced number of well-preserved available tissues. A summary of all macroscopical and histopathological ASF-associated [27] and bacterial-induced or background alterations [28,29,30,31] including immunohistochemistry results are shown in Table 3.

**Figure 2:**
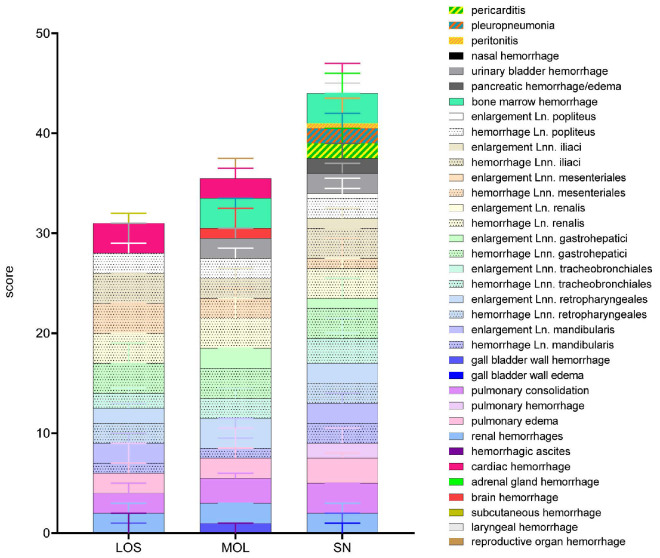
Summary of scoring results following macroscopical investigation of ASFV-infected wild boar carcasses from LOS, MOL and SN. Stacked bar diagram showing the total gross lesion score, which is composed of individual scores given for macroscopical findings shown on the right. Lesions were scored on a scale from 0 to 3. Bars indicate the median with range.

The overall score obtained upon macroscopical evaluation turned out to be the opposite when compared to the viral genome load and antigen score. Therefore, wild boar from SN tended to show a higher total score when compared to wild boar from LOS and MOL (Figure 2). Individual animal scores given for macroscopical findings are included in Supplementary Table S3.

In the following gross and histopathological findings, the different organ systems will be described.

### 3.3. Immune System

#### 3.3.1. Lymph Nodes

##### Gross Pathology

**Figure 3:**
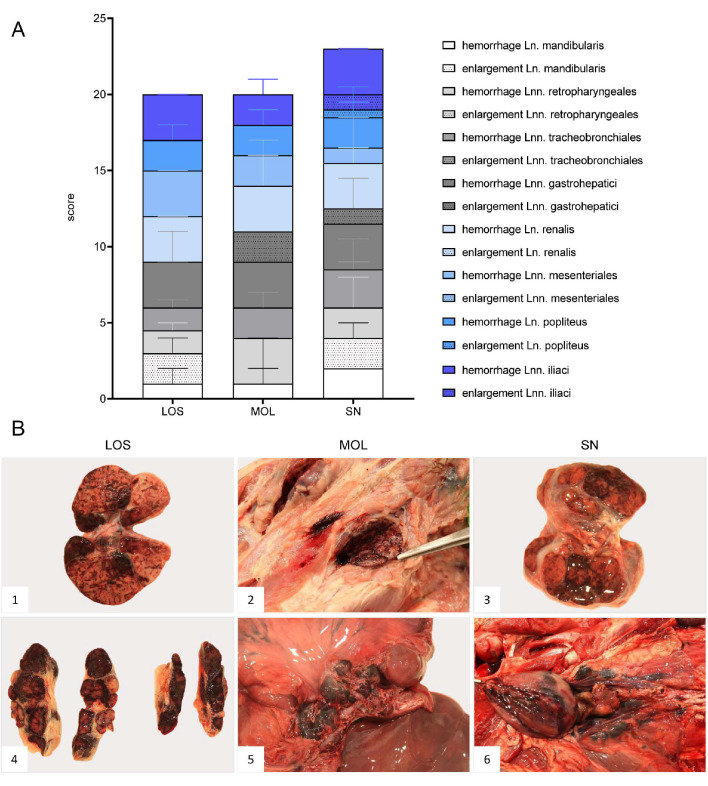
Representative macroscopical findings of lymph nodes in ASFV-infected wild boar carcasses from German outbreak areas. (**A**) Stacked bar diagram showing the total gross lesion score given for enlargement and hemorrhages of various lymph nodes evaluated on a scale from 0 to 3. Bars indicate the median with range per finding. (**B**) Lymph nodes (Ln. mandibularis ((**B1**)–(**B3**)), Ln. renalis (**B4**), Lnn. gastrohepatici (**B5**), Lnn. iliaci (**B6**)) revealed hemorrhages of varying degree.

In general, hemorrhagic lymphadenopathy was present in all animals irrespective of the outbreak area (Figure 3). Details are given in Supplementary File S1 and Table S3.

##### Histopathology

**Figure 4:**
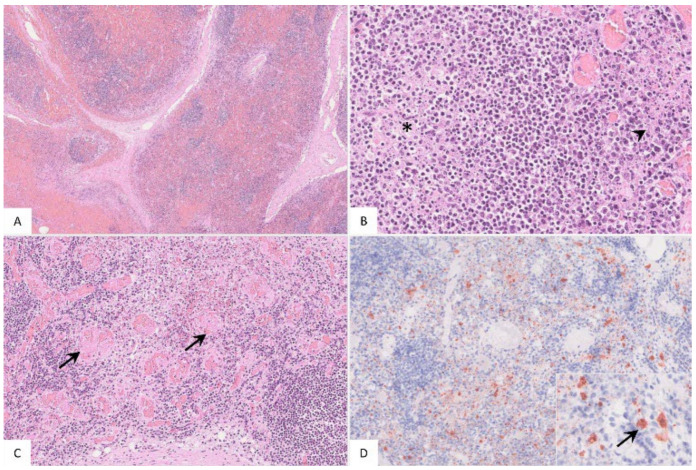
Pathohistological findings of the popliteal lymph node in German ASFV-infected wild boar carcasses. (**A**) Diffuse lymphoid depletion and hemorrhage affected the follicles, paracortex and medullary chords thereby effacing the physiological lymph node architecture, HE stain. (**B**) A lymphoid follicle with lymphoid depletion (asterisk) was surrounded by necrosis (arrowhead), HE. (**C**) Numerous vessels were occluded by fibrin thrombi (arrows) throughout the lymph node, HE. (**D**) A large number of viral-antigen-positive cells are shown, which were morphologically consistent with macrophages (inlay), anti-p72 immunohistochemistry, ABC method.

The popliteal lymph node was examined in more histological detail as demonstrated in Figure 4. The findings were characterized by lymphoid depletion, hemorrhages (Figure 4A,B), necrosis (Figure 4B) and vascular thrombosis (Figure 4C). Animals showed p72 positively labeled cells morphologically consistent with macrophages (Figure 4D). Details for individual animals are given in Table S2.

#### 3.3.2. Spleen

##### Gross Pathology

Macroscopic assessment of the spleen was limited due to poor preservation. Therefore, the spleen was evaluated by determination of the relative spleen weight based on a recent publication in domestic pigs [32] shown in Figure S1.

High relative spleen weight values were observed in all wild boar irrespective of the district of origin. Median values reached 0.81 (LOS), 0.69 (MOL) and 0.97 (SN).

##### Histopathology

Briefly, histological examination of the spleen revealed congestion and hemorrhage with lymphoid depletion in all wild boar (Figure S2A,B). Immunopositive cells were detected, phenotypically consistent with macrophages (Figure S2C,D). Individual histopathological results are summarized in Table S2.

#### 3.3.3. Bone Marrow

##### Gross Pathology

**Figure 5:**
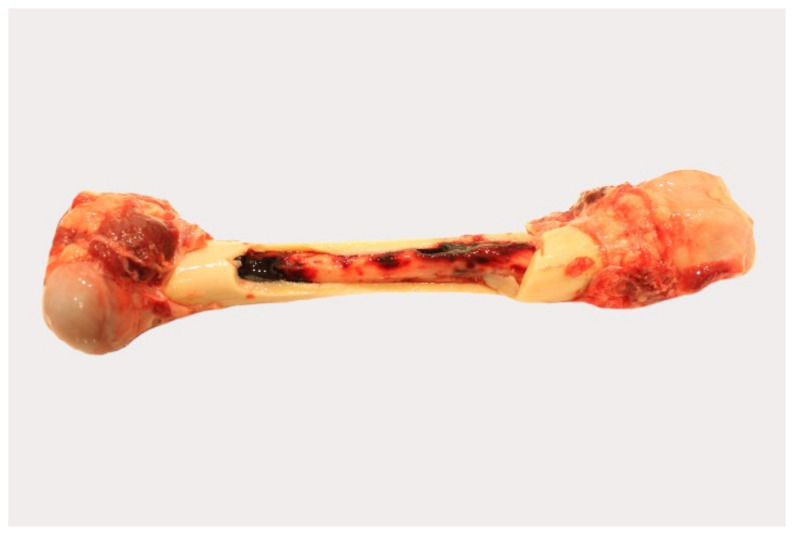
Gross pathology of the bone marrow of naturally ASFV-infected wild boar carcasses from German outbreak areas. Bone marrow hemorrhages, if present, were severe throughout.

Pathological changes in the femoral bone marrow included hemorrhages in all groups (Figure 5). All scores given for each individual animal can be found in Table S3.

Histopathological examination was not performed, because in the majority of animals, progression from red to yellow marrow had already occurred.

### 3.4. Respiratory System

#### 3.4.1. Lung

##### Gross Pathology

**Figure 6:**
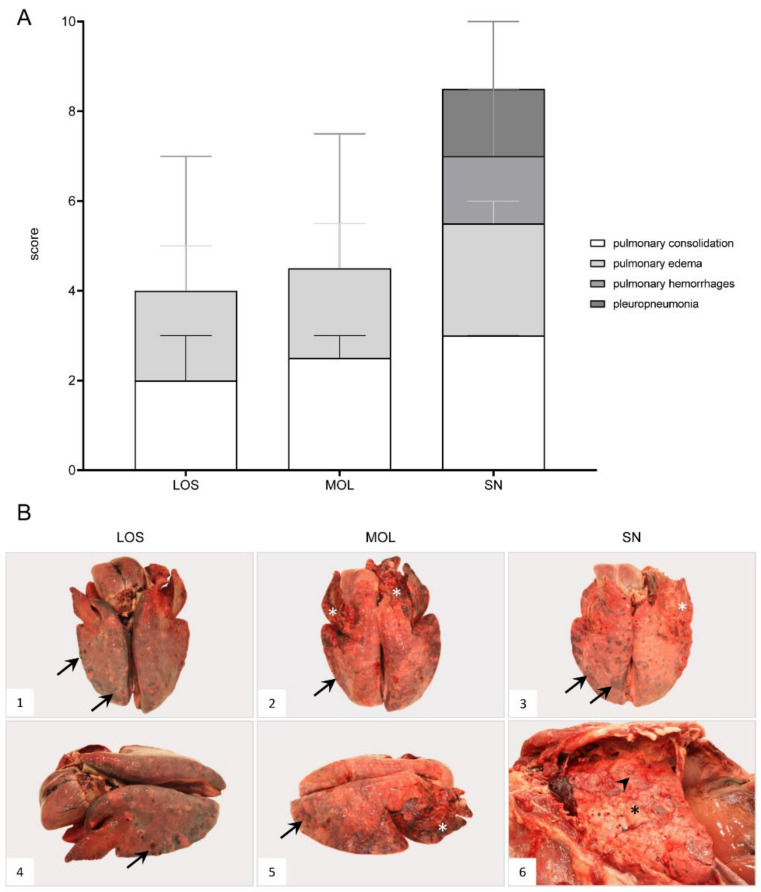
**Macroscopical lung lesions of ASFV-infected wild boar carcasses found in German districts**. (**A**) Stacked bar diagram demonstrating the median with range of individual scores given for each pathological criterion shown on the right legend. The presence and severity of each finding was scored from 0 to 3. (**B**) All lungs showed consolidated areas of different size (asterisk) and loss of pulmonary collapse ((**B1**)–(**B6**)). Pulmonary hemorrhages of varying severity are demonstrated by arrows ((**B1**)–(**B5**)). Chronic pleuropneumonia, likely due to bacterial infection, is shown in B6 with extensive fibrous pleural adhesions (arrowhead).

ASF-associated macroscopic findings of the lung were up to severe pulmonary edema, incomplete collapse with foci of consolidation and congestion as well as hemorrhages (Figure 6B, 1–4). In individual wild boar, fibrous pleuropneumonia, likely as a consequence of bacterial infection, was detected (Figure 6B, 5–6). Furthermore, isolated verminous pneumonia was present in wild boar from all districts. Details on macroscopic lung scores of all animals are summarized in Table S3 and described in Supplementary File S1.

##### Histopathology

**Figure 7:**
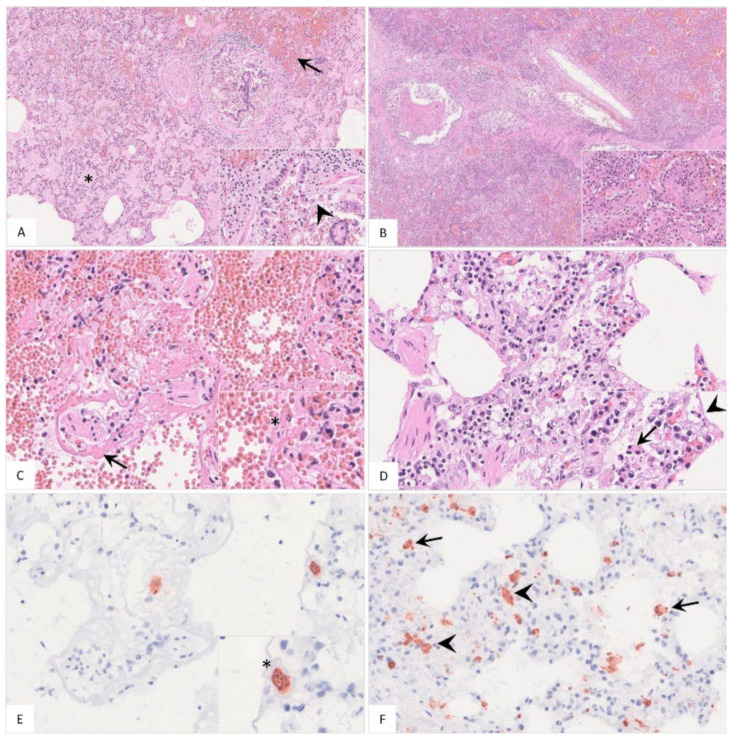
**Histopathological findings of lungs in German naturally ASFV-infected wild boar carcasses**. (**A**) Alveoli were filled with protein-rich edema fluid (asterisk), erythrocytes (arrow) and fibrin strands. The bronchiolus revealed epithelial necrosis (inlay, arrowhead) and contained cellular debris and erythrocytes. A distended pulmonary vein with fibrin thrombi was present left from the bronchiole, HE. (**B**) In a few animals, severe fibrino-suppurative to necrotizing bronchopneumonia was detected. Alveoli were densely filled with cellular debris, fibrin, viable and degenerate neutrophils, plasma cells, macrophages and lymphocytes as well as erythrocytes (inlay), HE. (**C**) A low number of wild boar showed loss of alveolar epithelium and hyaline membranes (arrow). An intravascular macrophage is indicated by asterisk (inlay), HE. (**D**) Alveolar septa showed epithelial necrosis (inlay, arrowhead), infiltration by necrotic macrophages (inlay, arrow), neutrophils, lymphocytes and plasma cells, HE. (**E**) and (**F**) Immunohistochemistry showed viral antigen-positive cells morphologically consistent with intravascular ((**E**), asterisk, consecutive section of (**C**)) intraalveolar ((**F**), arrow) and interstitial ((**F**), arrowhead) macrophages, anti p72-immunohistochemistry, ABC method.

Histopathological findings are shown in Figure 7 and Table S2. Pulmonary inflammation either presented as fibrino-suppurative to necrotizing bronchopneumonias, probably due to bacterial infections (Figure 7A–C), or interstitial pneumonia (Figure 7D). Positively labeled cells consistent with intravascular, -alveolar and interstitial macrophages were detected by immunohistochemistry (Figure 7E,F).

### 3.5. Cardiovascular System

#### 3.5.1. Heart

##### Gross Pathology

**Figure 8:**
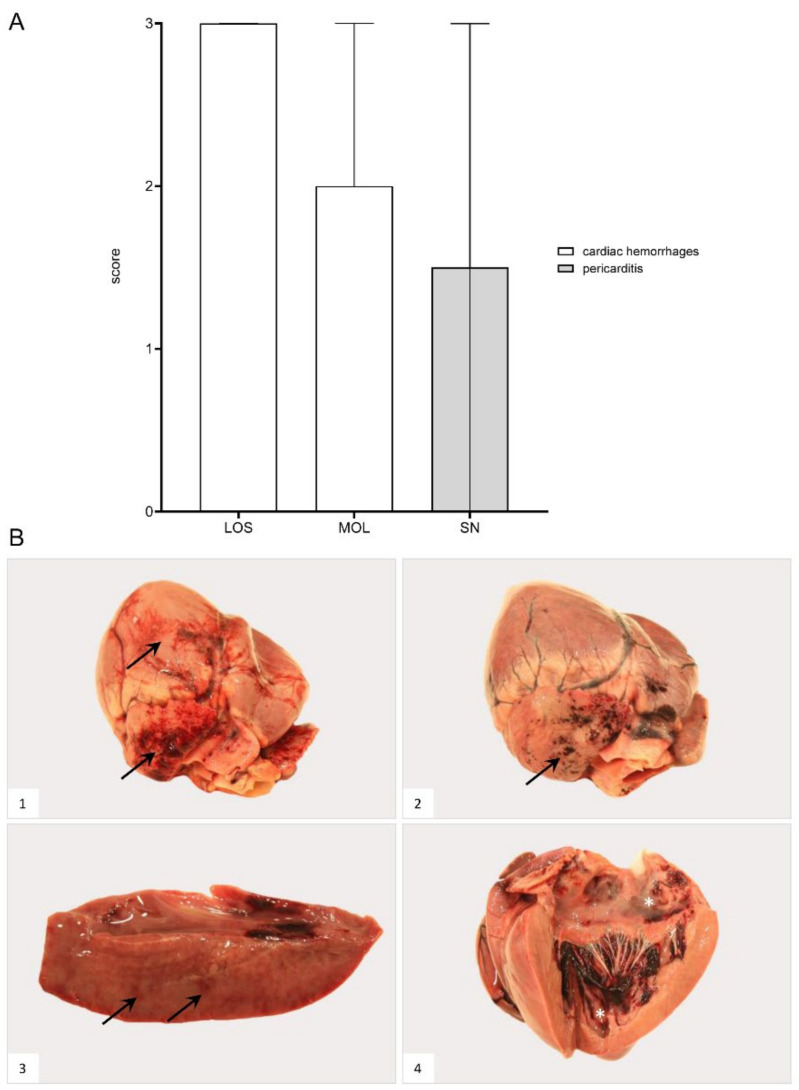
**Heart lesions in naturally ASFV-infected wild boar carcasses from German outbreak areas**. (**A**) Scoring of the heart included the presence and severity of hemorrhages as well as pericarditis, which were evaluated on a scale from 0 to 3. Bars indicate the median with range. (**B**) Hemorrhagic lesions of different locations and severity of ASFV-infected wild boar are shown. Multifocal paintbrush to coalescing hemorrhages were found in the epicardium (arrow) to a variable extent ((**B1**),(**B2**)). Scant myocardial hemorrhages (arrow) are indicated in (**B3**). Multifocal endocardial hemorrhages (asterisk) are present in (**B4**). The darker blood coagulum had to be differentiated from hemorrhages.

Hemorrhages affected wild boar of each group (Figure 8). Bacterial infection led to pericarditis with fibrous adhesions in one animal from SN. Details for individual animals are given in Supplementary File S1 and Table S3.

##### Histopathology

**Figure 9:**
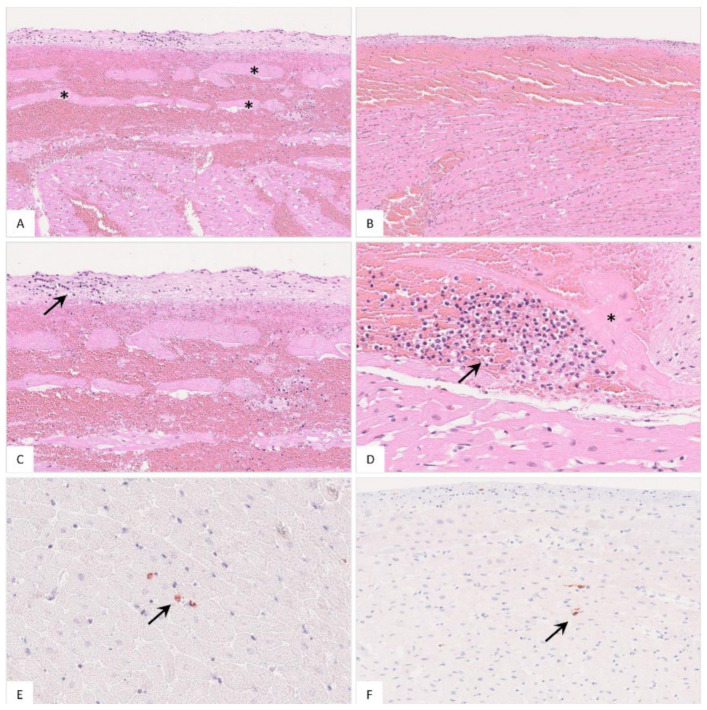
**Histopathology of the heart in naturally ASFV-infected wild boar carcasses from Germany**. (**A**) Massive hemorrhage involved the endocardium as well as the myocardium, displacing subendocardial Purkinje fibers (asterisk), HE. (**B**) The epicardium was also affected by diffuse hemorrhage radiating into the myocardium, HE. (**C**) Higher magnification from (**A**) shows minimal accumulation of infiltrating mononuclear cells in the endocardium (arrow), HE. (**D**) Subendocardial infiltrates (arrow) were also present between Purkinje fibers (asterisk), HE. (**E**,**F**) Immunohistochemistry of the heart showed only few positive macrophages (arrow), anti-p72 immunohistochemistry, ABC method.

In addition to hemorrhages (Figure 9A,B), in a few animals, there was endocardial and subendocardial infiltration by mononuclear cells (Figure 9C,D). Viral antigen was found in cells mostly morphologically consistent with macrophages (Figure 9E,F). Detailed histopathological evaluation of the heart can be found in Table S2.

### 3.6. Urinary System

#### 3.6.1. Kidney

##### Gross Pathology

**Figure 10:**
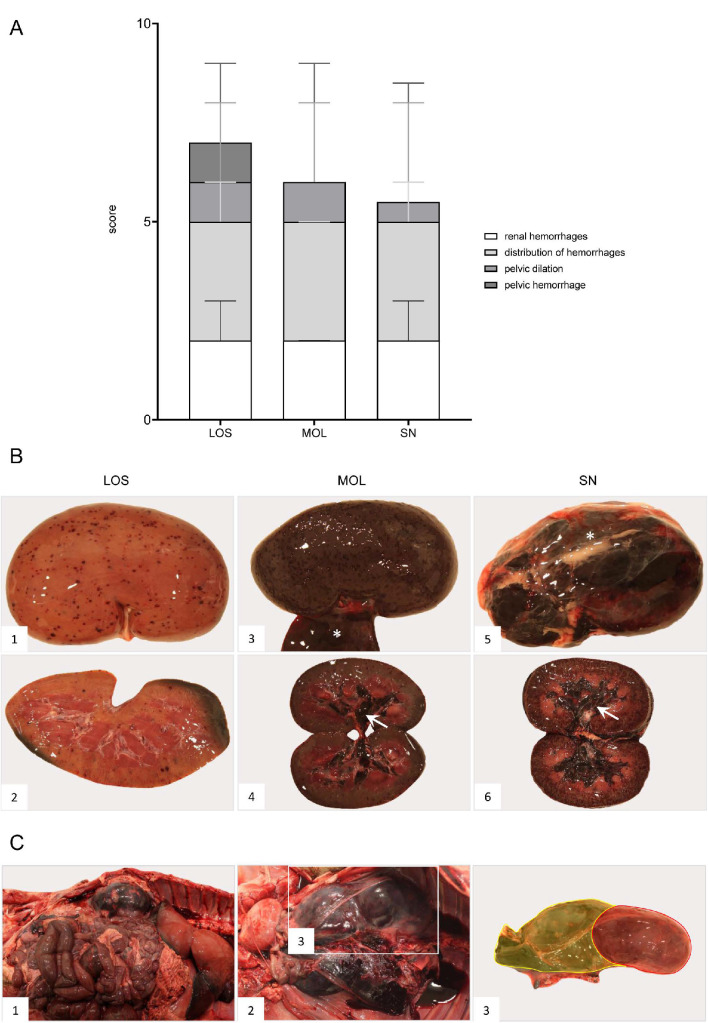
**Pathologic changes in kidneys of ASFV-infected German wild boar carcasses from different outbreak areas**. (**A**) Stacked bar diagram of gross lesion scoring of pathological criteria listed on the right. Scoring was conducted on a scale from 0 to 3 or from 0 to 4 (distribution pattern of hemorrhages). Individual scores are given as median values with range. (**B**) Hemorrhagic lesions of various size and severity affecting different parts of the organ are shown in ((**B1**)–(**B6**)). Multifocal petechiae with fewer ecchymoses primarily located to the renal cortex are depicted in ((**B1**),(**B2**)). Gray discoloration of the kidney periphery was due to beginning autolysis (**B2**). Mainly affecting the renal cortex (cortico-medullar pattern), diffuse ecchymoses are present in ((**B3**),(**B4**)). Marked dilation and diffuse bleeding into the renal pelvis are depicted in ((**B4**),(**B6**)) (arrows). To a lesser extent, oligofocal petechiae (arrowhead) could be found in the medulla (**B6**). Edema of the perirenal tissue is represented in ((**B3**),(**B5**)) (asterisk). (**C**) Massive hemorrhage resulted in expansion and bulging of the renal capsule ((**C1**),(**C2**)). The hemorrhage further extended into the perirenal and retroperitoneal tissue including the ureter (**C2**). To better distinguish the kidney and the extent of hemorrhage from (**C2**), the kidney was shaded red and the hemorrhage was highlighted in yellow (**C3**).

Renal and perirenal hemorrhages were present in all wild boar irrespective of the district (Figure 10). Details can be found in Supplementary File S1 and Table S3.

##### Histopathology

**Figure 11:**
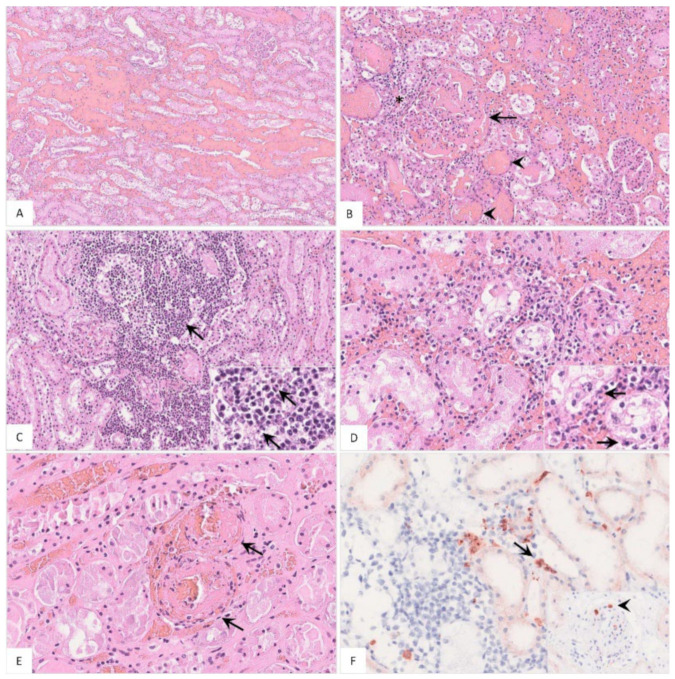
**Pathohistological findings of the kidney in naturally ASFV-infected wild boar carcasses.** (**A**) Diffuse hemorrhages were present expanding the renal medullary interstitium, HE. (**B**) A glomerulus showed extravasation of fibrin admixed with erythrocytes into the Bowman’s space (arrow). There was periglomerular infiltration of partly degenerated mononuclear cells (asterisk). Red blood cell casts were present in several tubules surrounding the glomerulus (arrowhead), HE. (**C**) Extensive mononuclear cell infiltrates accumulated around tubules and glomeruli (arrow) and revealed multiple foci of apoptosis/necrosis (inlay, arrow), HE. (**D**) In some areas, tubulointerstitial nephritis was associated with tubular epithelial apoptosis/necrosis (inlay, arrow), HE. (**E**) Fibrinoid vascular necrosis could be found in varying amounts of renal veins (arrow), HE. (**F**) Representative immunohistochemical image showing moderate numbers of positively labeled macrophages in the renal interstitium (arrow) or glomerular capillaries (inlay, arrowhead), anti-p72 immunohistochemistry, ABC method.

The histopathological findings included hemorrhages (Figure 11A), glomerular alterations (Figure 11B), non-suppurative tubulointerstitial nephritis, (Figure 11C), tubular epithelial necrosis (Figure 11D) and renal vein thrombosis (Figure 11E). Immunohistochemistry revealed positive cells morphologically consistent with macrophages (Figure 11F). Details on the histopathological findings are shown in Table S2.

#### 3.6.2. Urinary Bladder

##### Gross Pathology

**Figure 12:**
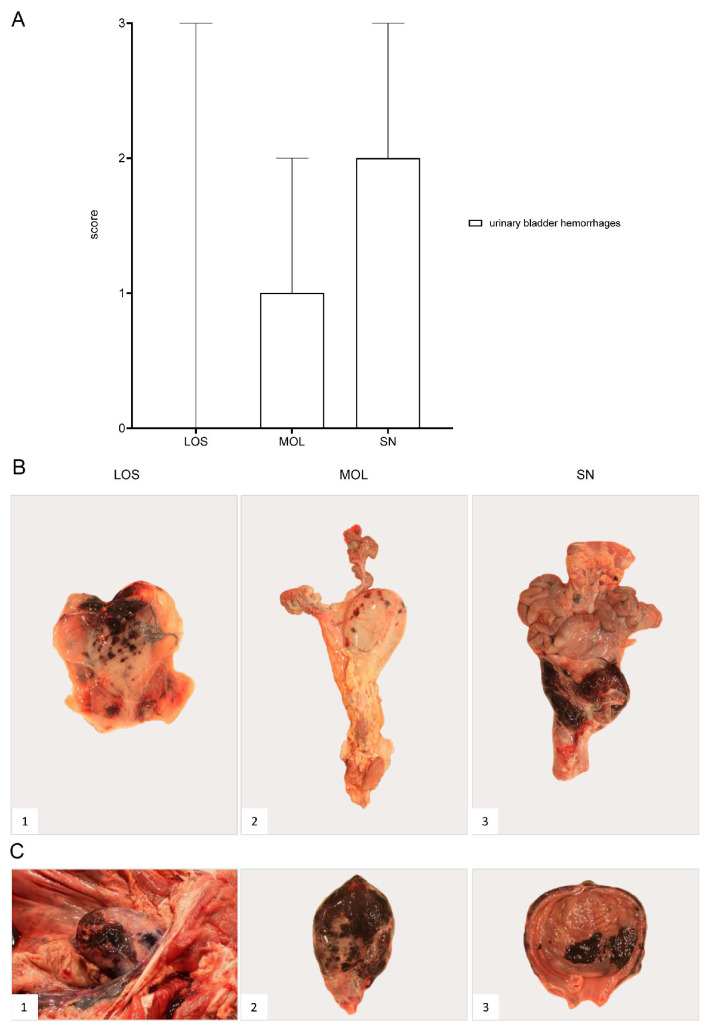
**Pathology of the urinary bladder in naturally ASFV-infected wild boar carcasses from German outbreak districts**. (**A**) Bar diagram showing hemorrhagic changes of the urinary bladder scored on scale from 0 to 3. Bars indicate the median with range. (**B**) Hemorrhages of varying severity were observed during necropsy. Multifocal-to-coalescing hemorrhages (**B1**) and multiple ecchymoses (**B2**) or severe, diffuse hemorrhage of the urinary bladder radiating into surrounding connective tissue (**B3**) were found. (**C**) Severe hemorrhages were located to the serosa ((**C1**),(**C2**)) as well as to the mucosal surface of the urinary bladder (**C3**).

The urinary bladder presented with hemorrhages in wild boar of all three groups (Figure 12). Details on the lesions found in the animals as well as individual scores can be found in Supplementary File S1 and Table S3.

Histopathological examination was not performed due to poor preservation.

### 3.7. Gastrointestinal System

#### 3.7.1. Liver and Gall Bladder

##### Gross Pathology

Due to poor preservation, not all livers could be examined. Hepatic congestion and hemorrhages as well as edema affecting the gall bladder wall were present (Figure S3). Details on lesions are given in Supplementary File S1 and Table S3.

##### Histopathology

Microscopical lesions of well-preserved livers included apoptosis/necrosis of Kupffer cells (Figure S4A) and hepatocytes (Figure S4B), and sinusoidal and periportal infiltrates (Figure S4C). Immunohistochemistry revealed positive immunolabeling of cells phenotypically consistent with Kupffer cells (Figure S4D). A summary of histopathological observations is included in Table S2.

#### 3.7.2. Stomach and Intestine

##### Gross Pathology

Due to progressive autolysis, the gastrointestinal tract could be evaluated only in individual animals. Macroscopic findings included hemorrhagic gastritis and hemorrhages in the small and large intestine as indicated in Figure S5. Hemorrhagic ascites was further detected. Occasionally, gastric ulcers as well as fibrous peritonitis, likely associated with bacterial infection, were also found in animals from SN. Supplementary File S1 and Table S3 provide detailed results.

Histopathological examination was not carried out due to advanced autolysis of the gastrointestinal tract.

### 3.8. Nervous System

#### 3.8.1. Brain

##### Gross Pathology

**Figure 13:**
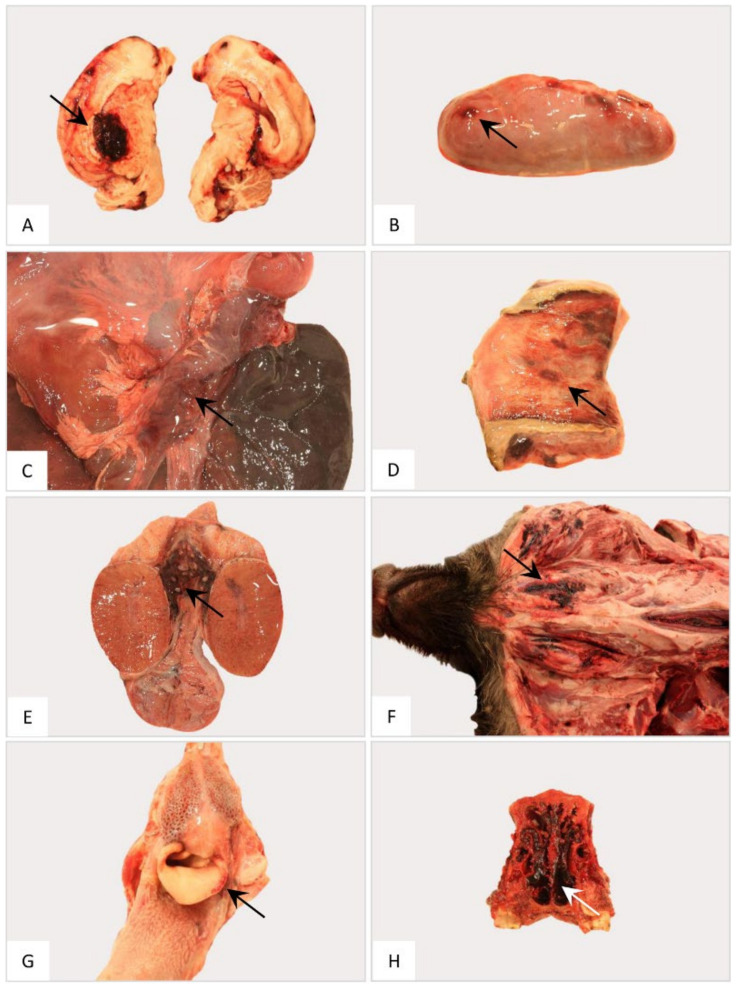
Gross pathology of the nervous, endocrine and reproductive organ systems and other findings in naturally ASFV-infected wild boar carcasses from Germany. Representative lesions included hemorrhages in the cerebrum (**A**), adrenal gland (**B**), pancreas (**C**), vestibulum vaginae (**D**), testis (**E**), subcutaneous tissue (**F**), larynx (**G**) and nasal mucosa (**H**). Arrows indicate hemorrhagic changes in the respective organs.

The brain was affected by hemorrhages only occasionally in some animals from MOL as shown in Figure 13A. Both the cerebellum and cerebrum were further evaluated by histopathology since data on respective lesions are sparse.

##### Histopathology

**Figure 14:**
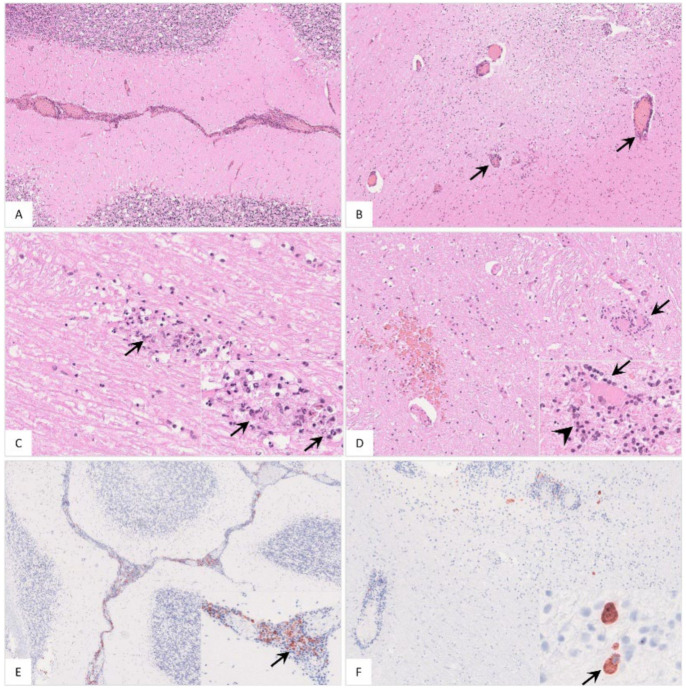
**Histopathological findings in the cerebellum of ASFV-infected wild boar carcasses**. (**A**) Meningitis was present in affected animals. (**B**) Cerebellar encephalitis was characterized by multifocal perivascular cuffs consisting of mononuclear cell infiltrates. (**C**) Parenchymal mononuclear infiltrates (arrow) showed multifocal apoptosis/necrosis (inlay, arrow). (**D**) Hemorrhage (left), perineural satellitosis (arrow, also see inlay) and microgliosis (inlay, arrowhead) were recognized. (**E**) and (**F**) Cerebellar meninges as well as brain parenchyma revealed positively labeled macrophages of differing amounts (inlays, arrow).

**Figure 15:**
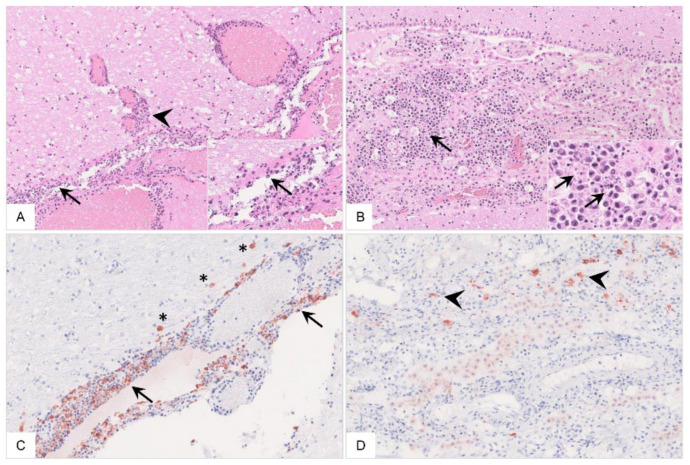
**Histopathology of the cerebrum of ASFV-infected wild boar carcasses.** (**A**) The meninges (arrow) and adjacent brain parenchyma (arrowhead) were infiltrated by mononuclear cells via Virchow Robin spaces. Mononuclear cells showed multifocal apoptosis/necrosis (inlay, arrow). Meningeal vessels were prominently dilated. (**B**) Mononuclear inflammation was limited to the choroid plexus within ventricles (arrow) with multifocal apoptosis/necrosis of infiltrating cells (inlay, arrow). There was degeneration of only a few plexus epithelial cells. (**C**,**D**) Immunopositive cells were present to variable extents in the meninges (arrow) and brain parenchyma (asterisk) as well as in the choroid plexus epithelium (arrowhead), phenotypically consistent with macrophages.

Microscopical findings of the cerebellum and cerebrum included meningitis, encephalitis and plexus choroiditis as depicted in Figure 14 and Figure 15, respectively. Occasionally, hemorrhage as well as satellitosis and microgliosis were detected. Detailed histopathological results are described in Supplementary File S1 and Table S2.

Immunohistochemical results showed viral antigen-positive cells with macrophage morphology.

### 3.9. Endocrine System

#### 3.9.1. Adrenal Gland

##### Gross Pathology

Hemorrhages were observed in the adrenal glands of animals from LOS and SN (Figure 13B).

##### Histopathology

**Figure 16:**
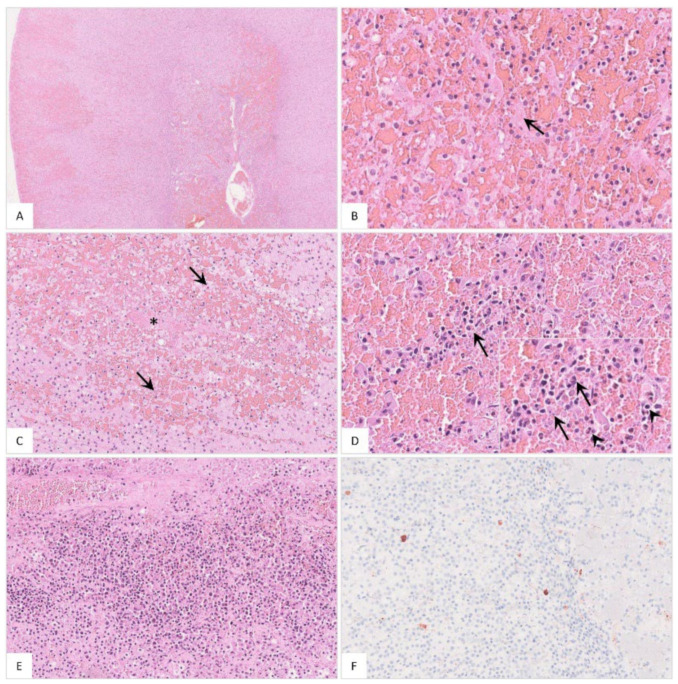
**Histopathological findings of the adrenal gland in ASFV-infected wild boar carcasses**. (**A**) Overview of the adrenal gland of a deceased wild boar. The adrenal gland showed extensive cortical and medullary hemorrhages. (**B**) Multifocally, fibrin thrombi were visible in the sinusoids (arrow). (**C**) Occasionally, areas of necrosis were present in the cortex (arrow). There was fibrin deposition (asterisk) and massive hemorrhage in the affected location. (**D**) The medulla was markedly expanded by hemorrhage. Infiltrating mononuclear cells as well as a few neutrophilic granulocytes (inlay, arrowhead) accumulated around degenerated cells (inlay, arrow). (**E**) The adrenal medulla was severely infiltrated by mononuclear cells admixed with fewer neutrophils. (**F**) Moderate amounts of antigen-positive macrophages were found in the majority of animals.

Histopathology revealed hemorrhages (Figure 16A), sinusoidal thrombosis and necrosis (Figure 16B,C) as well as inflammation (Figure 16D,E). Positively labeled macrophages were detected by immunohistochemistry (Figure 16F). Individual histopathological results are listed in Table S2.

#### 3.9.2. Pancreas

##### Gross Pathology

Pancreatic edema and hemorrhage were detected in animals from SN (Figure 13C). Histopathological examination was not carried out due to advanced autolysis.

### 3.10. Reproductive System

Occasionally, hemorrhages were found in the vaginal vestibulum in one wild boar from LOS (Figure 13D) and in the spermatic cord in a wild boar from MOL (Figure 13E).

### 3.11. Occasional Findings

Further hemorrhages were found in the subcutis in animals from LOS (Figure 13F), and in the epiglottis (Figure 13G) and nasal cavity (Figure 13H) in wild boar from SN.

### 3.12. Antibody Detection against African Swine Fever Virus

All animals were tested for anti-ASF antibodies by IPT as shown in Figure S6. Except for one animal from LOS, all wild boar developed antibodies of different titers between 200 and 800. Higher titers tended to be found in the animals from MOL, titers ranging from 200 to 1600, and in two wild boar from SN having titers of 800 and 3200, respectively. One animal from SN showed a titer of 40. In the fourth wild boar from SN, no test could be performed due to limited sample material.

For comparison, three domestic pigs from a previous study inoculated with the moderately virulent ASFV strain “Estonia 2014” were analyzed for anti-ASFV-specific antibodies. Starting at day 14 pi, all pigs developed antibody titers between 200 and 400. Since one pig had died at day 14 pi, only two animals could be analyzed in the following days. On day 21 pi, titers increased to 800 and 1600. On day 28 pi, titers further increased to 3200 or even remained at the same level of 1600 while on day 35 pi antibodies dropped in one animal to 800, but increased in the other pig to 3200. On day 41 pi, a second increase in the titer to 1600 was noted in one pig whereas in the other one antibodies remained constantly high at 3600.

## 4. Discussion

Filling the documentation gap on the pathology after ASF field infection, the aim of the present study was to examine ASFV-infected wild boar that succumbed to the disease under natural conditions in both virological and pathomorphological detail. Furthermore, the impact on the virulence of emerging virus variants II, III and IV in the ASF outbreak areas of Eastern Germany was analyzed.

A total of 16 wild boar aged between 0 and 2 years of different sexes were investigated. Despite the different preservation status, the organs of each animal could be examined for ASFV genome load and revealed consistently positive results. While it has to be noted that a direct comparison has to be conducted with great care due to many unknown factors, all animals were found as carcasses in affected regions and that would allow us to assume they reached a similar point of infection, i.e., the terminal phase. At that point, significant differences were not found between animals of different outbreak areas and the three variants, but wild boar from SN tended to show both lower viral genome loads and viral antigen scores compared to animals from LOS and MOL. However, the viral genome load has limited informative value at this point since viral genome can be detected up to 100 days after infection [33] and the time at which the genome load decreases varies greatly between experiments [11,18,19].

In addition to organ-wide detection of viral genome, all wild boar irrespective of the outbreak area and virus variant were diagnosed with characteristic and severe ASF lesions resembling a systemic hemorrhagic disease [6]. While no data exist for wild boar that died of ASF under natural conditions, pathology in domestic pigs has recently been described [21,34,35]. Typically, domestic pigs show comparable lesions such as hemorrhagic lymphadenopathy, splenomegaly, pulmonary consolidation and edema, hemorrhages in the heart and kidneys and hepatomegaly with edema of the gallbladder wall, as well as edematous, hemorrhagic meninges.

While most of the macroscopic findings in this study have been described after experimental infection in wild boar [36], they do not reflect the severity and diversity seen under field conditions. Comparing the three different virus variants, striking, but not significant, differences were evident. Interestingly, the highest total score for gross pathological changes was given for wild boar from SN infected with variant IV, followed by animals from MOL infected with variant III, which showed an intermediated total score, and wild boar from LOS infected with variant II, had the lowest macroscopical score.

For ASF, four different courses of the disease have been described and include peracute, acute, subacute and chronic stages, which are associated with typical lesions [6]. Petrov et al. [33] moreover specified the subacute stage as chronic-like and differentiated into lethal and transient course after infection with moderately virulent ASFV. Gross pathomorphological changes of the subacute/chronic-like stage include multifocal hemorrhages, edema, lymphadenitis, interstitial pneumonia and ascites [6,27,33] whereas bacterial secondary infections inducing fibrinous polyserositis, chronic pneumonia and necrosis of tonsils, however, without vascular changes, predominate in chronic courses [6]. Lesions in acutely and chronically ASFV-infected domestic pigs were also already presented in detail decades ago [12]. The animals with chronic disease showed comparable lesions as observed in the acutely infected pigs, but additionally revealed chronic changes particularly including pericarditis, pneumonia and lymphadenitis. In the present study, in contrast to the animals from LOS and MOL, although without statistical significance, wild boar from SN more frequently showed lesions most likely associated with bacterial infections indicative for a lethal subacute protracted disease course.

More specifically, chronic inflammatory processes such as fibrous pericarditis, pleuropneumonia and peritonitis were more frequently detected in SN animals. At the same time, wild boar from SN, and to a lesser extent also animals from MOL, tended to show more severe hemorrhages in the urinary bladder and bone marrow, but fewer acute hemorrhages as detected in the hearts of animals from LOS. Detailed pathomorphological investigation of experimentally infected wild boar that succumbed to highly virulent ASFV “Armenia07” infection revealed only mild petechiae of the urinary bladder, variable hemorrhages of the heart and congestion of the bone marrow while extensive hemorrhages or lesions induced by other circulating pathogens were absent [36].

Based on this, and in line with virological and immunohistochemical data, this may indicate that at least wild boar infected with the SN variant experienced a more protracted disease course than pigs from LOS suggesting a slightly decreased virulence of the SN virus variant IV to wild boar that still led to the death of the respective animals. This demonstrates that veterinarians, hunters and farmers need to be aware of less acute courses of ASF, usually attributed to classical swine fever, in order to consider this as important differential diagnosis in each case. However, considering the small number of carcasses and the indefinite sample material, this should be interpreted with caution and must be confirmed experimentally under standardized conditions in any case.

Although the majority of organs could be assessed macroscopically, we had to refrain from a detailed semiquantitative histopathological analysis because autolysis had already progressed too far in some cases, which would have considerably reduced the number of samples for investigation. However, in line with macroscopic findings, histopathology confirmed the severe course of disease in all animals regardless of the outbreak area and the virus variant. Since most wild boar studies focus only on macroscopic pathology, it is even more important to study the histopathology of natural ASF infection in more depth [17,18,36,37].

Most of the histopathological findings obtained in this study are fully comparable with those observed in domestic animals investigated upon outbreaks [21,34]. However, some of the observed lesions have already been described, but are not associated with ASF in the first line. For example, adrenal hemorrhages, which have been described to occur in wild boar upon experimental infection [36], were examined in more histopathological detail and revealed interesting results in the present study. Our findings mirror a condition known as Waterhouse Friderichsen syndrome [38]. It has been correlated with several bacterial and viral diseases and is characterized by severe hemorrhage, necrosis and microvascular thrombosis. Although the pathophysiology is not fully understood, hemorrhages are explained by a stress-induced release of adrenaline, vasculitis and coagulation disorders including disseminated intravascular coagulation. In line with the latter, microvascular thrombosis could be shown in multiple organs as signs of acute organ injury in wild boar investigated in this study [12].

Of note, histopathology further highlighted the unique finding of localized inflammation of the cerebral choroid plexus, which occurred in wild boar irrespective of the outbreak district, but mainly affected the majority of animals from MOL and SN. So far, there are only minor reports on ASF lesions in the central nervous system [12,34,39], which can occur at all stages of the disease as demonstrated by Moulton and Coggins [12] in acutely and chronically succumbing as well as in surviving pigs after experimental and natural infection. In addition to mononuclear infiltration of meningeal and cerebral vessels, perivascular hemorrhage, occasional vascular thrombosis and neuronal degeneration, necrosis of the choroid plexus epithelium has been described only once in a few acutely infected animals [12]. The naturally infected wild boar presented in this study showed pronounced mononuclear inflammation with massive cell deaths in addition to occasional necrosis of the plexus epithelium, again suggesting a longer disease course, at least in animals from SN.

To further extrapolate how long naturally infected wild boar might have lived with the disease, antibody titers were determined and compared to those of surviving ASFV “Estonia 2014” experimentally infected domestic pigs from a previous trial. In domestic pigs, low antibody titers were detectable from day 14 to a maximum titer of 400, then increased to a max of 3200 by day 28, and remained constantly high until 41 days post infection, at least in one domestic pig. However, the other pig showed a drop from 3200 to 800 on day 35 pi and a second subsequent increase. While it cannot be excluded that a consumption or decay of antibodies occurred, one should also consider measurement inaccuracies of the semiquantitative test when targeting the fluctuant antibody titers. When comparing this to wild boar, which showed titers of at least 200, the majority of animals independent of the outbreak area might have lived with ASF for more than 14 days.

As suspected, based on the pathological data in animals from SN, but also MOL, the course of the disease was probably longer since they tended to show higher titers of max 3200 and 1600, respectively, while wild boar from LOS reached titers of only max 800. Surprisingly, antibody titers showed no clear correlation to the chronicity of lesions observed in several wild boar since one animal from SN with obviously chronic lesions produced only minimal antibody titers. On the one hand, the chronic lesions in this animal could have already existed before and might not necessarily be associated with ASFV infection. On the other hand, as hypothesized above, the antibodies may have declined over time. To date, little is known about the host’s immune response against ASFV, but it is of general acceptance that antibodies directed against ASFV are not sufficient for protection against the disease [40]. However, experiments to investigate the dynamics of antibody development in ASF could be useful to draw conclusions on the disease in wildlife.

## 5. Conclusions

In summary, this is the first study describing the lesion spectrum in wild boar succumbing to ASF after infection with the different virus variants that have emerged within one year in Germany. Virological and pathomorphological data suggest possible differences in the virulence of the variants. At least, wild boar infected with the SN variant IV tended to experience a more protracted but nevertheless lethal disease course compared to animals infected with LOS variant II or the MOL variant III, which is more likely to be classified as intermediate. These findings are particularly important with regard to the spread and continued occurrence of the ASFV in endemic areas. To elucidate the pathogenicity and differences in the virulence and disease dynamics of the emerging virus variants more thoroughly, further experimental studies in wild boar as well as comparative investigations in domestic pigs under late human endpoint conditions are urgently needed. These studies should also address the impact of protracted disease courses on shedding and thus transmission characteristics.

## Figures and Tables

**Table 1:** Summary presentation of examined wild boar carcasses from three different German outbreak areas LOS, MOL and SN.

No	Origin	Virus Variant	Age (Year)	Sex	Weight (kg)	Stages of Decomposition *	Found Dead/Shot	Anomalies/Comments
1	LOS	II	<1	female	10	fresh stage	dead	Brachygnathia superior
2	LOS	II	<1	female	30	fresh stage	dead	/
3	LOS	II	>2	female	62	bloat stage	dead	/
4	LOS	II	<1	female	40	bloat stage	dead	/
5	LOS	II	<1	female	31	fresh stage	dead	/
6	LOS	II	<1	male	37	bloat stage	dead	/
7	LOS	II	<1	female	27	fresh stage	dead	/
8	MOL	III	<1	female	22	fresh stage	dead	/
9	MOL	III	<1	female	28	fresh stage	dead	/
10	MOL	III	<1	female	36	fresh stage	dead	/
11	MOL	III	<1	female	38	bloat stage	dead	/
12	MOL	III	<1	female	36	bloat stage	shot	Lung not available
13	SN	IV	<1	male	36	fresh stage	dead	/
14	SN	IV	<1	male	30	bloat stage	dead	Scavenger feeding marks (thorax)
15	SN	IV	<1	female	31	fresh stage	dead	/
16	SN	IV	>2	female	75	bloat stage	dead	/

* Stages of decomposition were classified as reviewed by Brooks [24] with further modifications. Fresh stage: no bloating, no discoloration. Bloat stage: bloating, gray to green discoloration of organs.

**Table 2:** Assessment of gross pathological criteria in ASFV-infected wild boar.

Organ	Macroscopic Finding	Annotations
Lymph node (popliteal)	Enlargement	/
	Hemorrhage	
Lung	Alveolar edema	/
	Interstitial edema	
	Hemorrhage	
	Collapse	
	Consolidation	
	Thoracic effusion	
	Pleuropneumonia	
Kidney *	Hemorrhage	Assessment of size (petechia, ecchymosis) and distributional pattern (focal (n = 1), oligofocal (n ≤ 20), multifocal (n ≥ 20))
	Pelvic dilation	/
	Pelvic hemorrhage	
Liver and gall bladder *	Congestion	/
	Gall bladder wall hemorrhage/edema	
Spleen *	Determination of relative spleen weight	/
Pancreas	Hemorrhage/edema	/
	Necrosis	
Abdominal cavity *	Peritonitis	/
	Ascitis	
Urinary bladder	Hemorrhage	/
Bone marrow	Hemorrhage	/
Heart	Hemorrhage	Describing localization: endocardial, myocardial, epicardial
	Pericardial effusion	/
	Pericarditis	
Tonsils	Hemorrhage	/
	Necrosis	
Brain	Hemorrhage	/
Adrenal gland		
Genitals		
Skin		
Larynx		

* Further lesions were described.

**Table 3:** Summary of macroscopical and microscopical lesions in ASF-infected wild boar carcasses. Pathological findings are listed as primary lesions, characteristically associated with ASF [27] and as lesions, usually induced by bacteria or as common background lesions [28,29,30,31].

Organs/Tissues	Gross Pathology	Histopathology	Immunohistochemistry
Immune system	**Primary lesions associated with ASF** Lymph nodes: • Hemorrhagic lymph-adenopathy Spleen: • Increased spleen weight Bone marrow: • Hemorrhages	**Primary lesions associated with ASF** Lymph nodes: • Lymphoid depletion • Thrombosis • Necrosis of interfollicular, paracortical areas and medullary chords Spleen: • Lymphoid depletion • Apoptosis/necrosis of myelomonocytic cells Bone marrow: • N/A	Lymph nodes: • Positive, macrophages Spleen: • Positive, macrophages Bone marrow: • N/A
Respiratory system	**Primary lesions associated with ASF** Lung: • Alveolar edema • Hemorrhages • Consolidation • Loss of collapse Nose: • Hemorrhages **Lesions, usually induced by bacteria or common background lesions** Lung: • Fibrous pleuropneumonia	**Primary lesions associated with ASF** Lung: • Alveolar edema • Hemorrhages • Necrotizing interstitial pneumonia Nose: • N/A **Lesions, usually induced by bacteria or common background lesions** Lung: • Fibrino-suppurative/necrotizing bronchopneumonia	Lung: • Positive, alveolar/interstitial macrophages Nose: • N/A
Cardiovascular system	**Primary lesions associated with ASF** Heart: • Hemorrhages (epi-, myo-, endocardial) **Lesions, usually induced by bacteria or common back-ground lesions** Heart: • Fibrous pericarditis	**Primary lesions associated with ASF** Heart: • Hemorrhages Mononuclear infiltration (endo-/subendocardial)	Heart: • Positive, macrophages
Urinary system	**Primary lesions associated with ASF** Kidney: • Hemorrhages (cortical, medullary, pelvic) • Perirenal edema and hemorrhages Urinary bladder: • Hemorrhages (mucosal, serosal, transmural)	**Primary lesions associated with ASF** Kidney: • Hemorrhages (interstitial, glomerular) • Vascular thrombosis Urinary bladder: • N/A **Lesions, usually induced by bacteria or common background lesions** Kidney: • Non-suppurative tubulointerstitial nephritis • Tubular necrosis	Kidney: • Positive, macrophages Urinary bladder: • N/A Urinary bladder: • N/A
Gastrointestinal system/abdominal cavity	**Primary lesions associated with ASF** Liver: • Congestion • Hemorrhages (subcapsular) Gall bladder: • Wall edema • Wall hemorrhages Stomach: • Hemorrhagic gastritis Small intestine: • Hemorrhages (serosal, mucosal) Large intestine: • Hemorrhages (serosal, mucosal) Abdominal cavity: • Hemorrhagic ascites **Lesions, usually induced by bacteria or common back-ground lesions** Stomach: • Ulcerative gastritis Abdominal cavity: • Fibrous peritonitis	**Primary lesions associated with ASF** Liver: • Apoptosis/necrosis of Kupffer cells and hepatocytes Gall bladder: • N/A Stomach: • N/A Intestine: • N/A **Lesions, usually induced by bacteria or common back-ground lesions** Liver: • Mixed-cellular sinusoidal and periportal infiltration	Liver: • Positive, Kupffer cells Gall bladder/stomach/intestine: • N/A
Nervous system	**Primary lesions associated with ASF** Brain: • Hemorrhages	**Primary lesions associated with ASF** Brain: • Hemorrhages • Non-suppurative meningitis (cerebral, cerebelar) • Non-suppurative encephalitis (cerebral, cerebellar) • Non-suppurative plexus choroiditis	Brain: • Positive, macrophages
Endocrine system	**Primary lesions associated with ASF** Adrenal gland: • Hemorrhages Pancreas: • Hemorrhages • Edema	**Primary lesions associated with ASF** Adrenal gland: • Hemorrhages (cortical, medullary) • Sinusoidal thrombosis • Mixed-cellular infiltration (medullary Pancreas: • N/A	Adrenal gland: • Positive, macrophages Pancreas: • N/A
Reproductive system	**Primary lesions associated with ASF** Testicle (spermatic chord): • Hemorrhages Vestibulum: • Hemorrhages	• N/A	• N/A

## Data Availability

All data available can be obtained on request from the corresponding authors.

## Supplementary Materials

The following supporting information can be downloaded at: https://www.mdpi.com/article/10.3390/pathogens11111386/s1, Table S1: Summary of individual organ genome copy numbers in wild boar; Table S2: Summary list of histopathological changes and immunohistochemistry results in wild boar; Table S3: Summary list of gross lesions scored on a semiquantitative scale in wild boar; Figure S1: Relative spleen weights of naturally ASFV-infected wild boar, Figure S2: Histopathology of the spleen of naturally ASFV-infected wild boar carcasses, Figure S3: Macroscopical findings of the liver in German ASFV-infected wild boar carcasses, Figure S4: Histopathological results detected in the liver of naturally ASFV-infected wild boar carcasses from Germany, Figure S5: Gross pathology of the gastrointestinal tract in naturally ASF-infected wild boar carcasses from German outbreak areas, Figure S6: Antibody titers determined by immunoperoxidase test in German wild boar carcasses compared to experimentally infected domestic pigs on different days pi, File S1: Detailed analysis.
