# Supplementary material for: Pathology of African Swine Fever in Wild Boar Carcasses Naturally Infected with German Virus Variants
Source: Pathogens. 2022 Nov 20;11(11):1386. doi: 10.3390/pathogens11111386 (PMC9699169; doi:10.3390/pathogens11111386)
Supplement: Supplementary file 1 [file pathogens-11-01386-s001.zip › Supplemental figure legends.pdf]

Supplemental figures

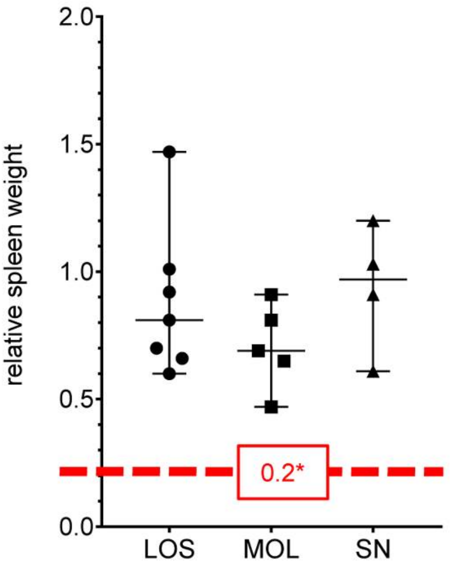

Figure S1. Relative spleen weights of naturally ASFV-infected wild boar. Dot plot showing the relative spleen weight values obtained from ASFV-infected wild boar from German districts LOS, MOL and SN. The median with range is indicated by error bars. *As a reference value, the mean relative spleen weight of healthy domestic food-producing pigs of 0.2 was used according to a recent publication (27).

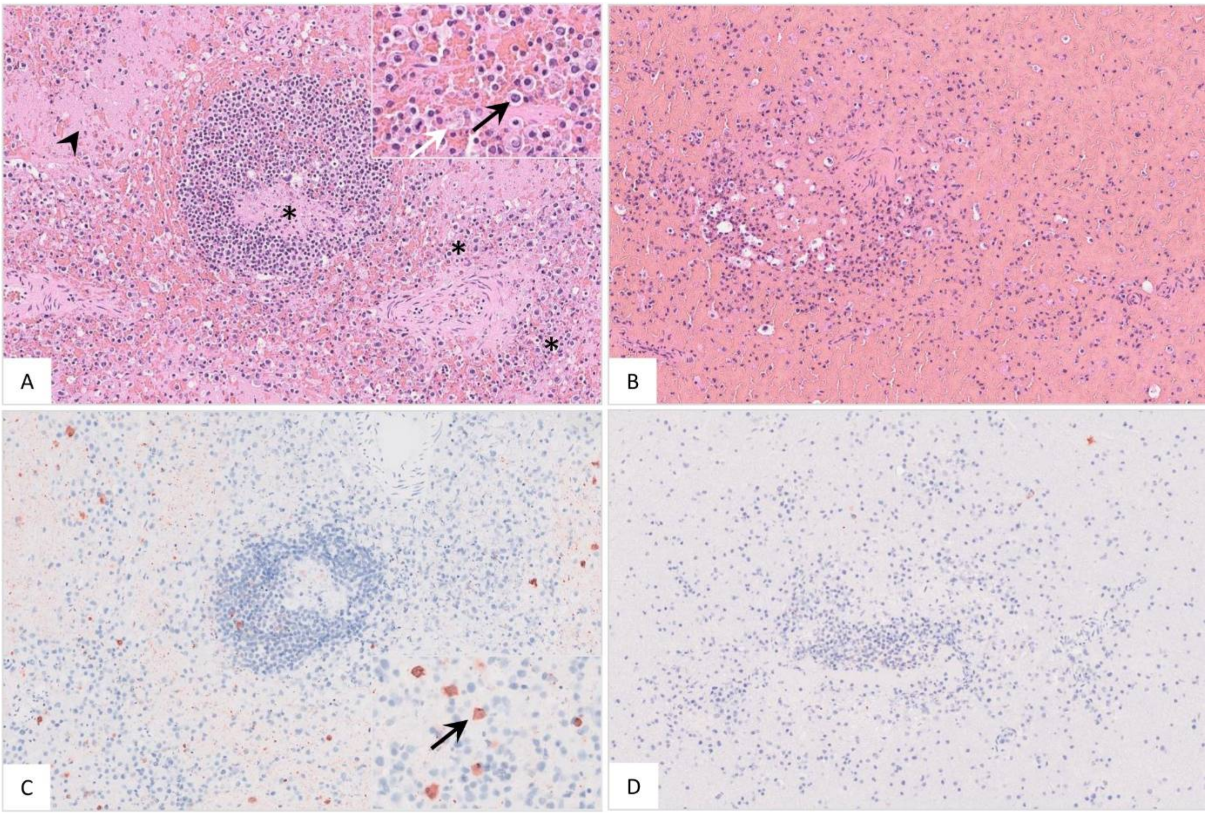

Figure S2. Histopathology of the spleen of naturally ASFV-infected wild boar carcasses. (**A**) A lymphoid follicle and periarteriolar lymphoid sheaths showed lymphoid depletion (asterisk) with enlarged splenic ellipsoids (arrowhead). Red pulp macrophages were hypertrophic or swollen (black arrow) and degenerate (white arrow) (inlay), HE. (**B**) Splenic architecture was replaced by necrosis and hemorrhage, HE. (**C**) Mainly moderate, but also

(**D**) low numbers of antigen-positive cells phenotypically consistent with macrophages (C, inlay, arrow) were present in the spleens, anti p72-immunohistochemistry, ABC method.

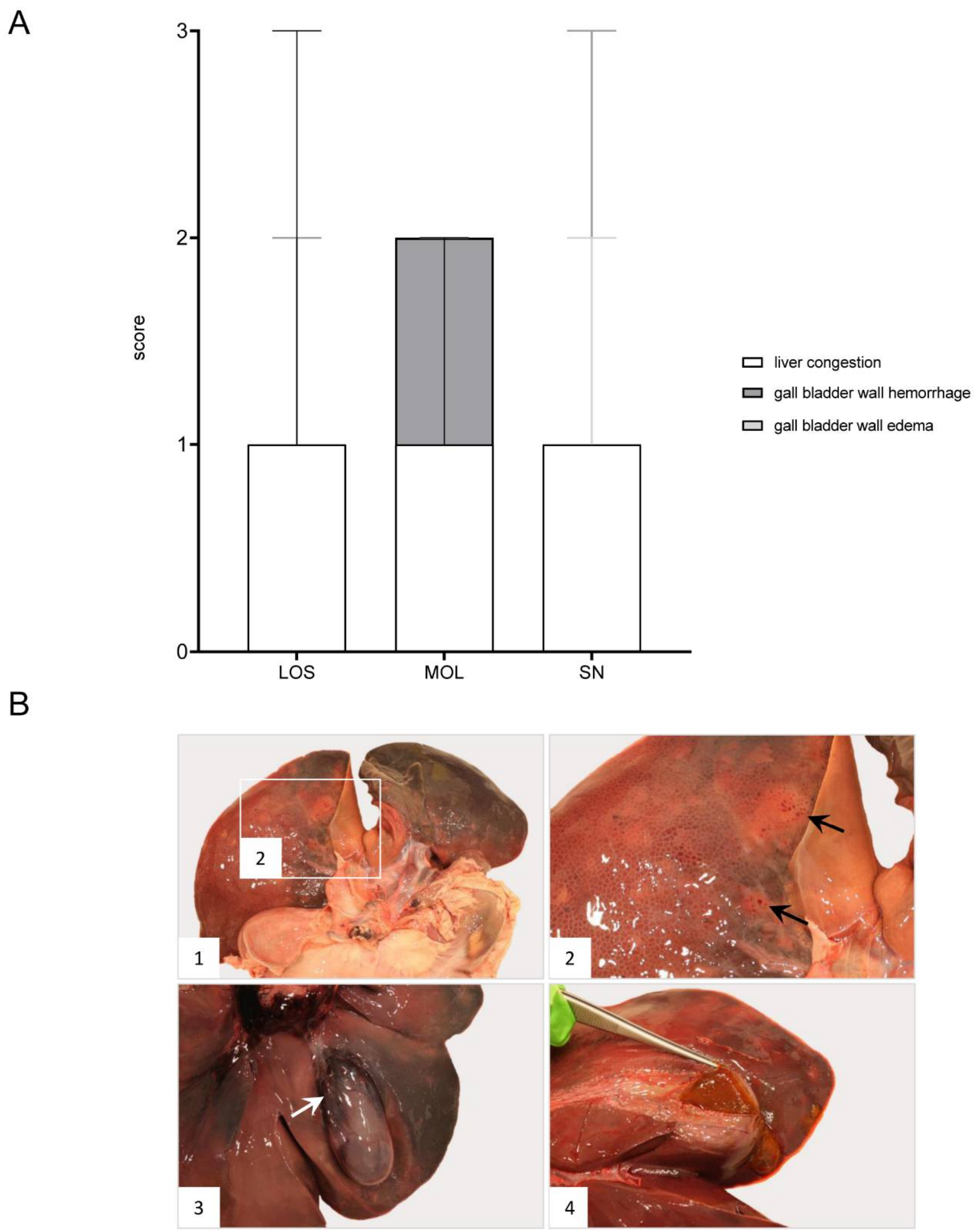

Figure S3. Macroscopical findings of the liver in German ASFV-infected wild boar carcasses. (**A**) Stacked bar diagram of gross scoring of hepatic congestion as well as hemorrhage and edema affecting the gall bladder wall on a scale from 0 to 3. Scores are presented as the median value with range. (**B**) Representative macroscopical liver lesions are shown in B1–4. Generally, livers were consistently autolytic to varying degrees (B1–4). In a single wild boar, multifocal subcapsular petechiae (arrows) were observed (B1–2). Coalescing hemorrhages of a mildly edematous gall bladder were detected (B3). Viscous biliary sludge was detectable throughout in wild boar (B4).

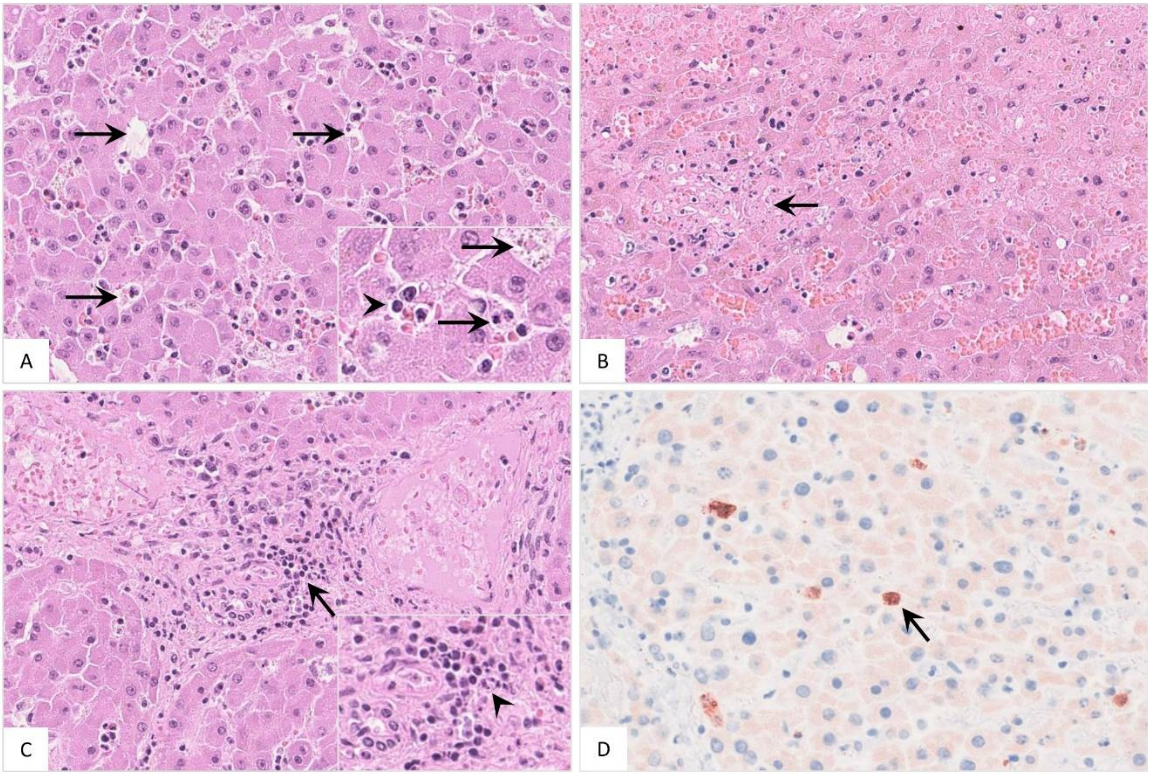

Figure S4. Histopathological results detected in the liver of naturally ASFV-infected wild boar carcasses from Germany. (**A**) The liver revealed moth-eaten appearance (arrow) due to multifocal apoptosis/necrosis of Kupffer cells (inlay, arrows). The sinusoids were mildly infiltrated by small numbers of neutrophils (inlay, arrowhead). (**B**) In contrast to Kupffer cells, only a few hepatocytes showed apoptotic/necrotic changes (arrow). (**C**) There was mild periportal infiltration of mononuclear cells (arrow), which showed cellular degeneration (inlay, arrowhead). (**D**) Mostly moderate numbers of positively labeled Kupffer cells (arrow) were detected.

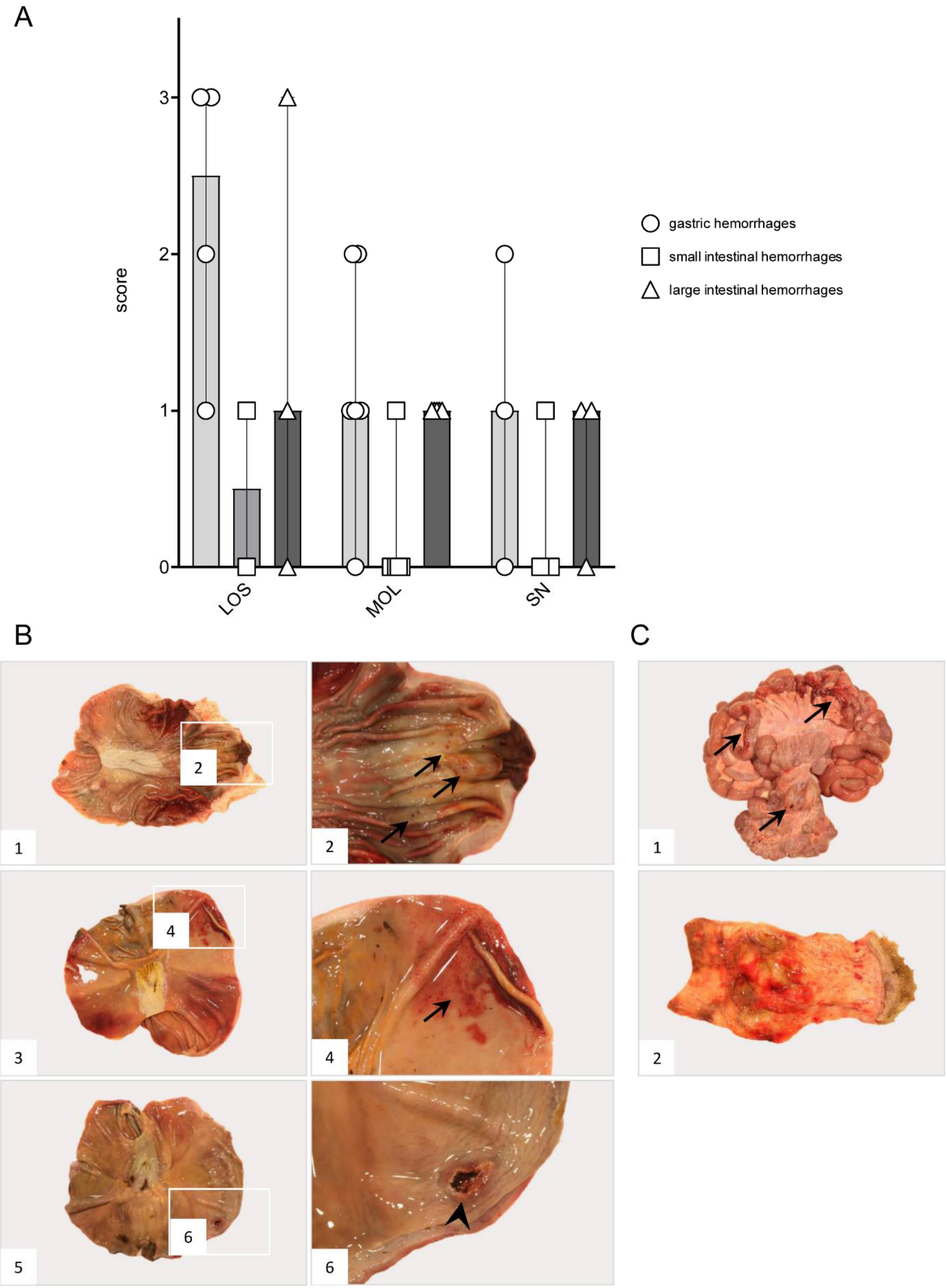

Figure S5. Gross pathology of the gastrointestinal tract in naturally ASF-infected wild boar carcasses from German outbreak areas. (**A**) Stacked bar diagram demonstrating the median values with range of criteria investigated in wild boar. The gastrointestinal tract was investigated for serosal and mucosal hemorrhages, if not already autolytic. Hemorrhages of the stomach and small and large intestines were scored from 0 to 3. (**B**) Pathological alterations in the stomach were found in almost all wild boar, which were possible to investigate. Multifocal-to-diffuse petechiae (arrows) were found (B1–4). Multiple chronic ulcers of the gastric mucosa (arrowhead) were detected in an animal

from the SN district (B5–6). (**C**) Paintbrush hemorrhages (arrows) were sporadically observed on the small and large intestinal serosa (C1), while multifocal-to-diffuse rectal mucosal hemorrhages occurred more frequently (C2).

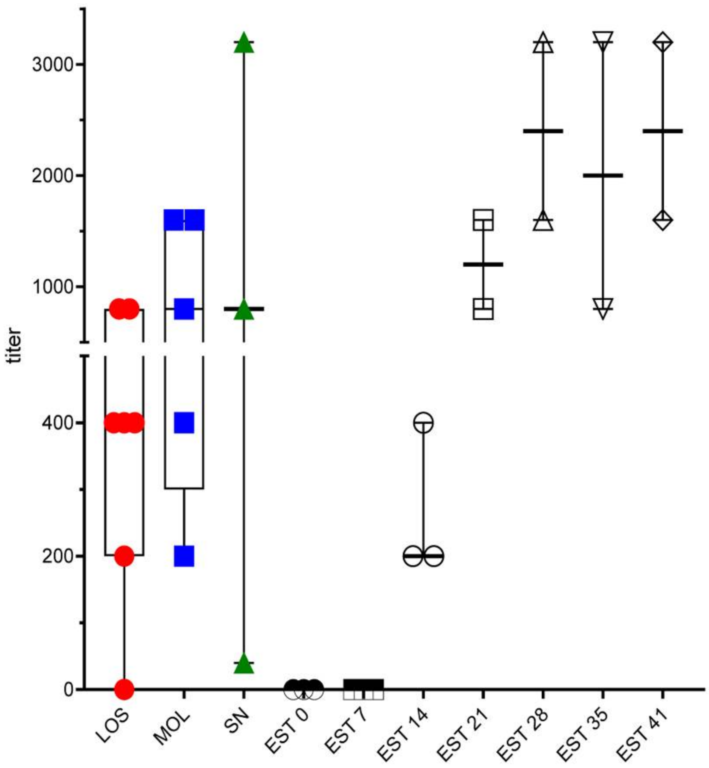

Figure S6. Antibody titers determined by immunoperoxidase test in German wild boar carcasses compared to ASFV "Estonia 2014" experimentally infected domestic pigs on different days pi. LOS = Landkreis Oder-Spree, MOL= Märkisch-Oderland, SN= Spree-Neiße, EST= Estonia 2014.
