# Supplementary material for: Pathology of African Swine Fever in Wild Boar Carcasses Naturally Infected with German Virus Variants
Source: Pathogens. 2022 Nov 20;11(11):1386. doi: 10.3390/pathogens11111386 (PMC9699169; doi:10.3390/pathogens11111386)
Supplement: Supplementary file 1 [file pathogens-11-01386-s001.zip › Supplemental material 1.pdf]

Supplementary Material 1—Detailed Analysis

Immune System

Lymph Nodes

Gross Pathology

Wild boar originating from the LOS district showed mild to severe enlargement and hem-orrhages, especially of the gastrohepatic and renal lymph nodes followed by mesenteric and iliac nodes. Similar, but generally moderate to severe lesions mainly affecting the gastrohepatic, renal and iliac nodes were observed in wild boar from the MOL and SN area.

Respiratory System

Lung

Gross Pathology

Briefly, in wild boar from LOS, moderate to severe pulmonary alveolar edema was present in 5/7 animals. In all wild boar incomplete retraction collapse with consolidation of varying severity was found. Congestion was present in all wild boar while widespread hemorrhages were observed in 3/7 animals. In one animal, severe, focal, fibrinous, chronic pleuropneumonia likely caused by bacterial secondary infection was evident in the caudal lobe.

Lung lesions could be assessed in 4/5 MOL animals since one boar was killed by a lung shot. In three animals, alveolar edema was detected of moderate to severe degree. The same animals showed loss of retraction pulmonary collapse whereas variable consolida-tion was present in all four affected animals. Two wild boar showed severely consolidated areas, one of these animals further had marked, chronic fibrous pleuropneumonia of the right lung due to bacterial secondary infections. While congestion varied between the an-imals, severe hemorrhages were found in two wild boar.

Similar lesions occurred in wild boar found dead in SN. Up to severe alveolar edema was present in all animals. The lung of one wild boar was severely autolytic making any further evaluation impossible. In the remaining three pigs, signs of pulmonary

inflamma-tion were evident. All lungs lacked retraction pulmonary collapse and consolidation was severe in two wild boar. All lungs were severely congested, but extensive hemorrhages were found only in two animals. Bacterial secondary infections presented as Ssevere, chronic fibrinous pleuropneumonia of the right lung was present in two animals.

Cardiovascular System

Heart

Gross Pathology

Severe hemorrhages were more frequently seen in 5/7 wild boar found in the LOS district. The epicardium was affected in five, the myocardium in three and the endocardium in four animals.

Comparable severe lesions were present in 3/5 wild boar from MOL. Changes were observed in the epi- and endocardium in two animals whereas the myocardium was additionally affected in another wild boar.

In contrast, only 1/4 wild boar from the SN area showed widespread bleeding located to the epi- and myocardium. Extensive, chronic fibrous pericarditis most likely with bacterial involvement was further detected in two wild boar from SN.

Urinary System

Kidney

Gross Pathology

Two wild boar from LOS showed focal or multifocal petechiae, three revealed multifocal to diffuse ecchymoses and another two had diffuse hemorrhages throughout the organ. A cortical-medullar pattern was observed in four animals whereas in three pigs hemor-rhages were equally distributed in the cortex and medulla. Dilation of the renal pelvis with few hemorrhages was present in four animals. In one animal severe perirenal edema was present.

Renal hemorrhages were detected in 4/5 wild boar from MOL. Three animals showed diffuse ecchymoses, one revealed multifocal petechiae. Except for one animal that pre-sented a cortical-medullar pattern, hemorrhages occurred equally in the cortex

and me-dulla. In two animals there was marked dilation of the renal pelvis with diffuse hemor-rhages. Perirenal edema and hemorrhages of marked severity were found in one wild boar.

Renal lesions were observed in 3/4 wild boar from SN. Multifocal to diffuse ecchy-moses were detected in two animals whereas in one pig the whole kidney was affected by diffuse hemorrhage. The cortex was more severely affected than the medulla. Mild to se-vere pelvic dilation in two wild boar with additional diffuse hemorrhages in another an-imal were seen. In one pig hemorrhages extended to the perirenal tissue massively dilat-ing the renal capsule and periureteral tissue.

Urinary Bladder

Gross Pathology

Multifocal to coalescing hemorrhages of the urinary bladder wall were present in one wild boar found in the LOS district while all other animals revealed no lesions.

More frequently, hemorrhagic lesions in the urinary bladder occurred in 3/5 wild boar cadavers from MOL. One wild boar displayed very few mucosal petechia while the two others presented with mottled hemorrhages of moderate severity.

In 3/4 animals originating from the SN district urinary bladder hemorrhages were most severe. Two out of three animals showed large coalescing areas of hemorrhages af-fecting the serosa and the mucosa. Milder lesions were observed in the other animal.

Gastrointestinal System

Liver and Gall Bladder

Gross Pathology

Due to poor preservation of the liver one animal from LOS had to be excluded from investigation. Congestion of the liver was severely present in two and mildly present in four wild boar. Multifocal subcapsular hemorrhages were observed in one wild boar. While none of the wild boar showed edema of the gall bladder wall, slight hemorrhages were found in a single animal.

Only three livers were evaluated from MOL due to progressive autolytic processes. Mild to moderate congestion was apparent in two livers. Gall bladder lesions could be as-sessed in 4/5 MOL animals and included mild hemorrhages, but no edematous changes.

All wild boar from SN showed mild congestion of the liver with 1/4 animal revealing a mildly edematous gall bladder wall with moderate hemorrhages.

Wild boar of all districts showed highly viscous bile that was defined as biliary sludge.

Stomach and Intestine

Gross Pathology

In 4/7 animals from LOS the stomach could be evaluated while advanced autolysis prohibited investigation of other animals. In these animals, hemorrhagic gastritis of dif-fering severity was detected. The small intestine showed progressive autolysis in five pigs. In one out of the two well preserved intestines, serosal multifocal pinpoint hemorrhag-espetechiae were found. As observed for the small intestine, also the large intestine was severely autolytic. Here, only in 2/7 pigs multifocal to diffuse hemorrhages were found on the serosal and mucosal rectal surface. Hemorrhagic ascites was detected in one animal.

In 4/5 animals from MOL mainly mild hemorrhagic gastritis was present. One animal revealed multifocal hemorrhages in the small intestinal serosa whereas all wild boar had multifocal hemorrhages in the large intestine, especially affecting the rectal mucosa. In 2/5 wild boar hemorrhagic ascites was present.

Due to progressive autolysis of the gastrointestinal tract of only 3/4 animals from SN was investigated. In two wild boar mild or moderate hemorrhagic gastritis, respectively, was present. Chronic ulcerative gastritis was found in one animal. Multifocal serosal hemorrhages were detectable in one wild boar whereas hemorrhages in the large intestine occurred in two animals mainly affecting the rectal mucosa. Two wild boar revealed chronic fibrinous fibrous peritonitis.

Brain

Histopathology

In the majority of animals originating from LOS, inflammatory processes were found in both the cerebellum and cerebrum. Inflammation of the cerebellum was found in 3/7 animals while the cerebrum was affected in 6/7 animals. Cerebellar meningitis was pre-sent in three animals, two of them had additional encephalitis. Cerebral meningitis was found in five animals while encephalitis was less frequent and occurred only in two wild boar. However, three animals showed inflammation limited to the plexus choroideus. Hemorrhages in the cerebrum were found in two animals.

In contrast to animals from LOS, all pigs from MOL showed cerebellar and/or cerebral inflammation, respectively. Whereas meningitis was detected in all wild boar, en-cephalitis was diagnosed in four pigs. One wild boar additionally showed cerebellar parenchymal hemorrhage. As the cerebellum, the cerebrum was affected by meningitis in four animals, by encephalitis and by plexus choroiditis in three pigs. Hemorrhagic changes were seen in one wild boar.

In animals found in SN, the cerebellum showed changes in 3/4 animals while the cerebrum was affected in all boar. Only one pig showed cerebellar meningeal inflamma-tion while three animals displayed cerebellar encephalitis. Similar findings were obtained in the cerebrum with meningitis noted in one and plexus choroiditis found in all animals. Neither the cerebellum nor the cerebrum showed any hemorrhagic changes.

Generally, inflammatory infiltrates mainly consisted of large numbers of partly degenerate mononuclear cells including macrophages, lymphocytes and some plasma cells. Immunohistochemical results varied widely among animals, ranging from a low to a large amount of viral antigen-positive cells showing macrophage morphology. In three animals from SN, in one from MOL and in one from LOS the cerebellum was immuno-histochemically negative. In the cerebrum, antigen was detected in all pigs except for one animal from LOS.
