# Supplementary material for: Pathology of African Swine Fever in Wild Boar Carcasses Naturally Infected with German Virus Variants
Source: Pathogens. 2022 Nov 20;11(11):1386. doi: 10.3390/pathogens11111386 (PMC9699169; doi:10.3390/pathogens11111386)
Supplement: Supplementary file 1 [file pathogens-11-01386-s001.zip › Table S2.pdf]

Histopathology

Table S2. Summary list of histopathological changes and immunohistochemistry results

			No.															
**Organ**	**Microscopic finding**	**Annotations**	**1**	**2**	**3**	**4**	**5**	**6**	**7**	**8**	**9**	**10**	**11**	**12**	**13**	**14**	**15**	**16**
**District**			LOS	LOS	LOS	LOS	LOS	LOS	LOS	MOL	MOL	MOL	MOL	MOL	SN	SN	SN	SN
**Virus variant**			II	II	II	II	II	II	II	III	III	III	III	III	IV	IV	IV	IV
Lymph node (popliteal)	Lymphoid depletion																	
	Congestion/ hemorrhage																	
	Necrosis	Paracortex																
		Medullary chords																
	Vascular thrombosis																	
	Viral antigen		3	3	2	3	2	3	3	2	3	3	3	3	3	2	1	2
Spleen	Lymphoid depletion																	
	Necrosis red pulp			A	A	A	A	A	A	A	A	A		A	A	A		
	Congestion/ hemorrhage										A			A		A		
	Viral antigen		3	2	1	2	0	3	2	1	3	2	2	2	2	3	1	2
Lung (cranial lobe)	Alveolar edema				A													
	Hemorrhage				A													A
	Bronchopneumonia	Fibrino-suppurative, necrotizing			A													A
	(broncho)-interstitial pneumonia	fibrino-necrotizing			A													A
	Viral antigen		3	2	2	2	1	3	2	2	3	3	2	3	3	1	1	3
Kidney	Nephritis	Tubulointerstitial, mononuclear cells			N/A								A	A		A		A
	Hemorrhage	Cortical			N/A								A	A		A		A

		Medullary			N/A								A	A		A		A
		Pelvic			N/A								A	A		A		A
	Vascular thrombosis				N/A								A	A		A		A
	Viral antigen		2	2	N/A	3	0	3	2	1	2	3	1	1	2	2	1	2
Liver	Kupffer cell apoptosis/necrosis			A	A	A		A	A	A	A	A	A	N/A		A		
	Hepatocyte apoptosis/necrosis			A	A	A		A	A	A	A	A	A	N/A		A		
	Sinusoidal infiltrates	Neutrophils		A	A	A		A	A	A	A	A	A	N/A		A		
		Mononuclear cells		A	A	A		A	A	A	A	A	A	N/A				
	Periportal infiltrates	Mononuclear cells		A	A	A		A	A	A	A	A	A	N/A		A		
		Eosinophils		A	A	A		A	A	A	A	A	A	N/A		A		
	Viral antigen		2	2	2	2	1	3	2	1	2	3	2	N/A	1	1	1	2
Heart	Hemorrhages	Endocardial		N/A		N/A		N/A			N/A		A	N/A	N/A	N/A	N/A	
		Myocardial											A	N/A				
		Epicardial		N/A	N/A		N/A					N/A	A	N/A				
	Endocardial infiltration	Mononuclear cells		N/A	N/A			N/A			N/A		A	N/A	N/A	N/A	N/A	
	Viral antigen		2	0	2	2	0	1	1	0	2	1	1	N/A	2	1	1	2
Cerebellum	Meningitis																	
	Encephalitis																	
	Hemorrhage																	
	Viral antigen		1	3	1	2	0	1	1	0	3	3	1	1	0	0	0	1
Cerebrum	Meningitis	Mononuclear cells																
	Encephalitis	Mononuclear cells																
	Choroiditis	Mononuclear cells																
	Hemorrhage																	
	Viral antigen		1	3	1	2	0	3	2	1	2	3	3	1	1	1	1	1

Adrenal gland	Hemorrhage	Cortical		A	N/A			A	A	A		A	A	A		A		A
		Medullary		A	N/A			A	A	A		A	A	A		A		A
	Sinusoidal thrombosis			A	N/A			A	A	A		A	A	A		A		A
	Necrosis	Cortical		A	N/A			A	A	A		A	A	A	A	A		A
		Medullary		A	N/A			A	A	A		A	A	A		A		A
	Adrenalitis	Mononuclear cells mixed with neutrophils		A	N/A			A	A	A		A	A	A		A		A
	Viral antigen		2	2	N/A	2	1	3	2	2	3	2	2	2	2	2	2	2

Histopathological changes are indicated as present or not present, A = autolysis, evaluation not possible or N/A = not available. The viral antigen amount was determined on a semiquantitative scale: (+) = ≤ 3 positive cells; (++) = 4-15 positive cells; (+++) = ≥ 16 positive cells.
