# Supplementary material for: Pathology of African Swine Fever in Wild Boar Carcasses Naturally Infected with German Virus Variants
Source: Pathogens. 2022 Nov 20;11(11):1386. doi: 10.3390/pathogens11111386 (PMC9699169; doi:10.3390/pathogens11111386)
Supplement: Supplementary file 1 [file pathogens-11-01386-s001.zip › Table S3.pdf]

Gross pathology

Table S3. Summary list of gross lesions scored on a semiquantitative scale

			No.															
**Organ**	**Macroscopic finding**		**1**	**2**	**3**	**4**	**5**	**6**	**7**	**8**	**9**	**10**	**11**	**12**	**13**	**14**	**15**	**16**
**District**			LOS	LOS	LOS	LOS	LOS	LOS	LOS	MOL	MOL	MOL	MOL	MOL	SN	SN	SN	SN
Lymph node (popliteal)	Enlargement		0	0	0	0	0	0	0	0	0	0	0	1	0	0	2	1
	Hemorrhage		3	0	2	3	2	2	2	2	2	2	2	2	2	0	3	2
Lung	Alveolar edema		2	3	0	0	1	3	2	3	3	0	2	1	3	3	2	2
	Hemorrhage		0	0	0	3	3	3	0	3	3	0	0	N/A	3	0	3	0
	Retraction		1	2	2	1	2	2	1	1	2	0	1	N/A	1	1	1	2
	Consolidation		3	3	1	2	3	1	2	3	3	1	2	N/A	3	A	3	1
	Pleuropneumonia		3	0	0	0	0	0	0	3	0	0	0	N/A	3	0	0	3
Kidney	Hemorrhage	Size	2	1	3	2	2	1	3	2	2	1	0	2	2	0	2	3
		*Distribution	4	1	4	3	3	3	4	3	3	3	0	3	4	0	3	3
	Pelvic dilation		0	1	3	1	0	0	3	3	0	1	1	3	3	3	3	1
	Pelvic hemorrhage		0	1	3	1	1	0	0	0	3	3	0	0	3	0	0	0
Liver	Congestion		3	1	A	1	3	1	1	0	2	1	A	A	1	1	1	1
	Gall bladder wall edema		0	0	A	0	0	0	0	0	0	0	0	A	0	0	1	N/A
GIT	Hemorrhages	Stomach	2	1	N/A	N/A	3	N/A	3	1	1	1	0	2	2	N/A	1	0
		Small intestine	A	A	A	A	1	A	0	0	1	0	0	0	0	A	1	0
		Large intestine	A	A	3	A	1	A	0	1	1	1	1	1	1	A	1	0
Urinary bladder	Hemorrhages		0	0	0	0	3	0	0	1	2	2	0	0	3	1	3	0
Bone marrow	Hemorrhages		0	0	0	3	3	0	3	3	3	3	3	3	3	2	3	3
Heart	hemorrhages	Endocardial	0	0	3	3	3	0	3	3	0	3	0	0	0	0	0	0
		Myocardial	0	0	3	0	3	3	0	3	0	0	0	0	0	0	3	0
		Epicardial	0	0	3	3	3	3	3	0	2	3	0	0	0	0	3	0

Lesions were scored on a semiquantitative scale; score 0 = normal, score 1 = mild, score 2 = moderate and score 3 = severe. *The distributional pattern of kidney hemorrhages was determined from 0 to 4; 0 = normal, 1 = focal, 2 = oligofocal, 3 = multifocal, 4 = diffuse. A = autolysis, evaluation not possible; N/A = not available.
