# Supplementary figures and images for: Pathology of African Swine Fever in Wild Boar Carcasses Naturally Infected with German Virus Variants
Source: Pathogens. 2022 Nov 20;11(11):1386. doi: 10.3390/pathogens11111386 (PMC9699169; doi:10.3390/pathogens11111386)

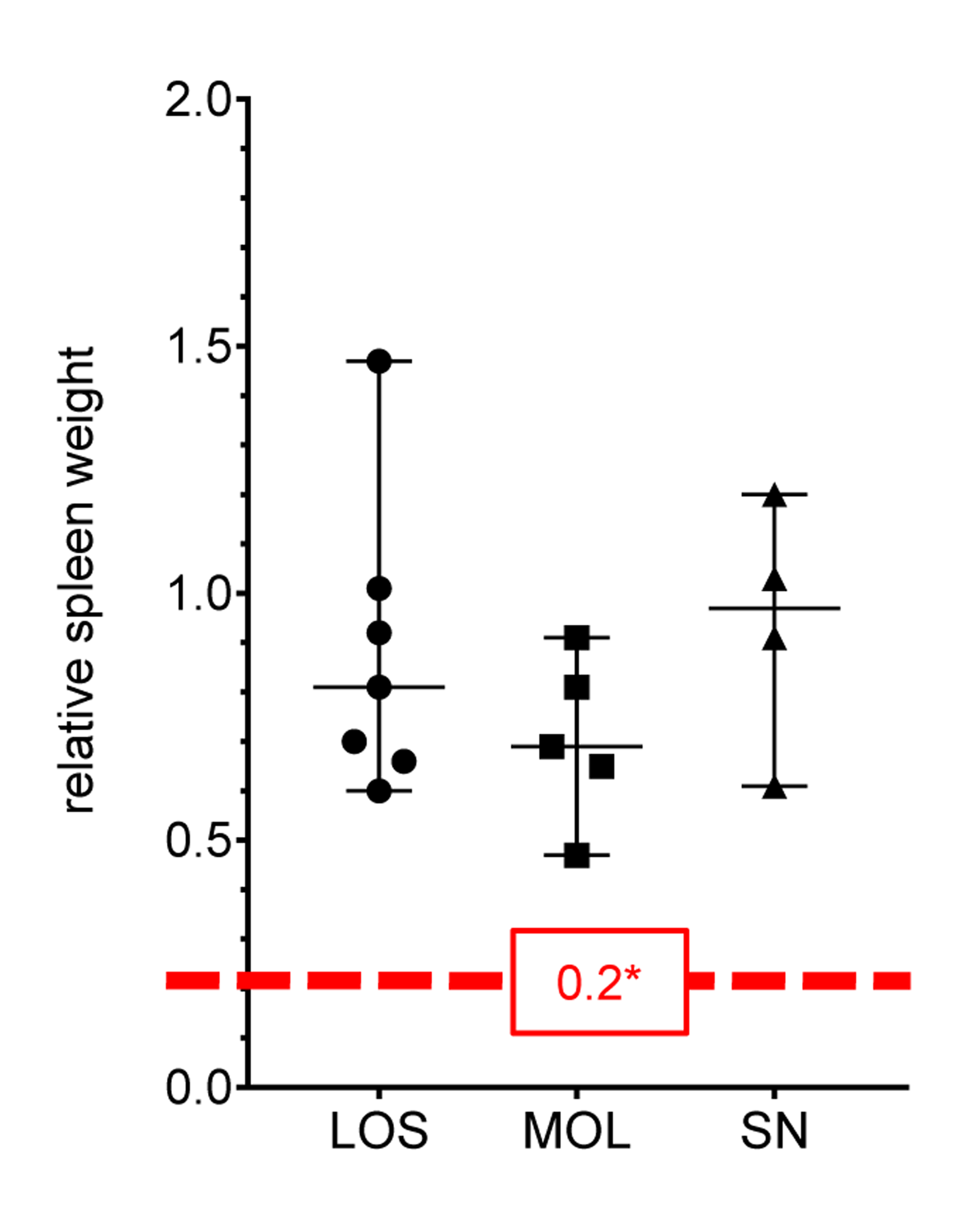

Supplement: Supplementary file 1 [file pathogens-11-01386-s001.zip › Fig S1.tif]

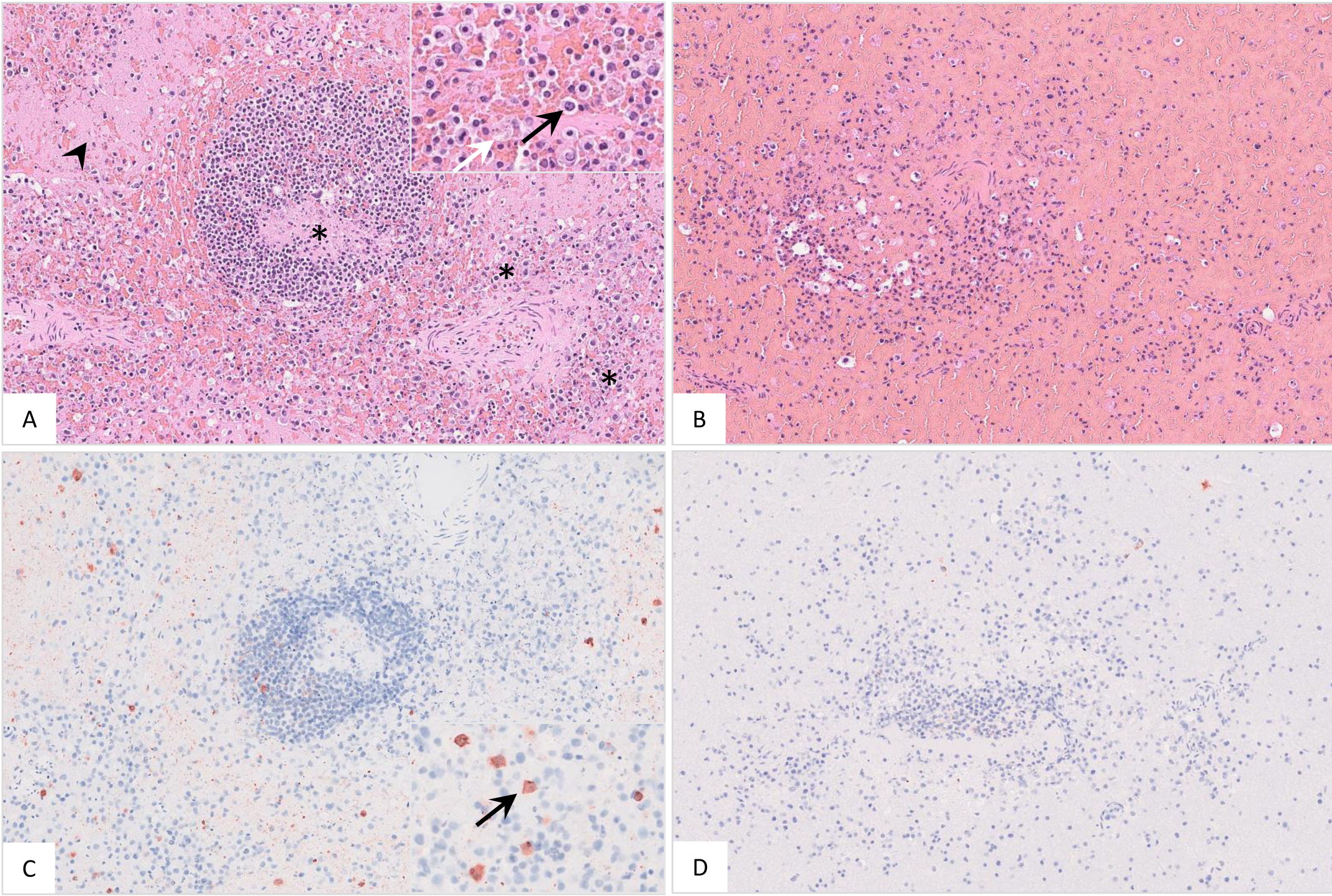

Supplement: Supplementary file 1 [file pathogens-11-01386-s001.zip › Fig S2.tif]

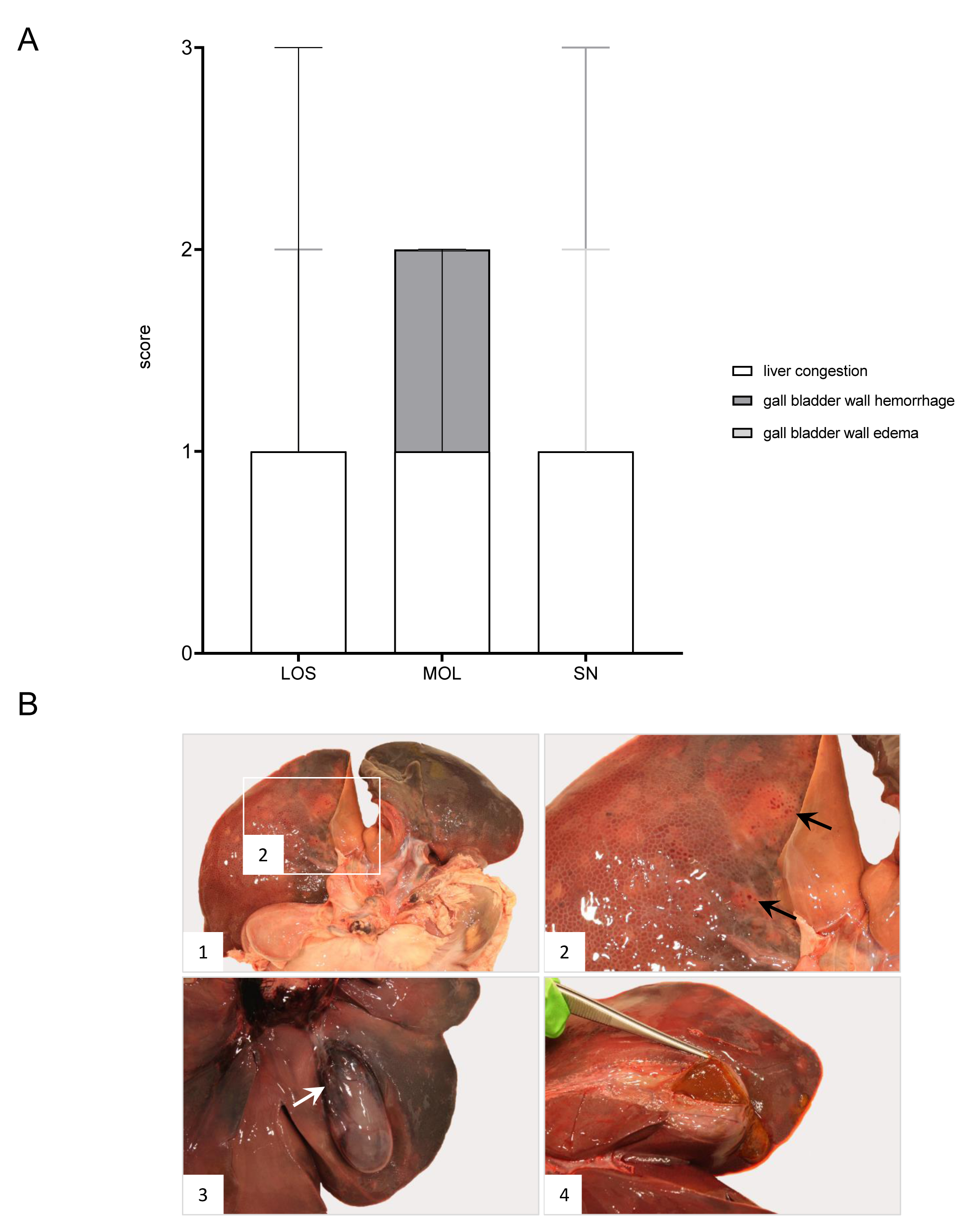

Supplement: Supplementary file 1 [file pathogens-11-01386-s001.zip › Fig S3.tif]

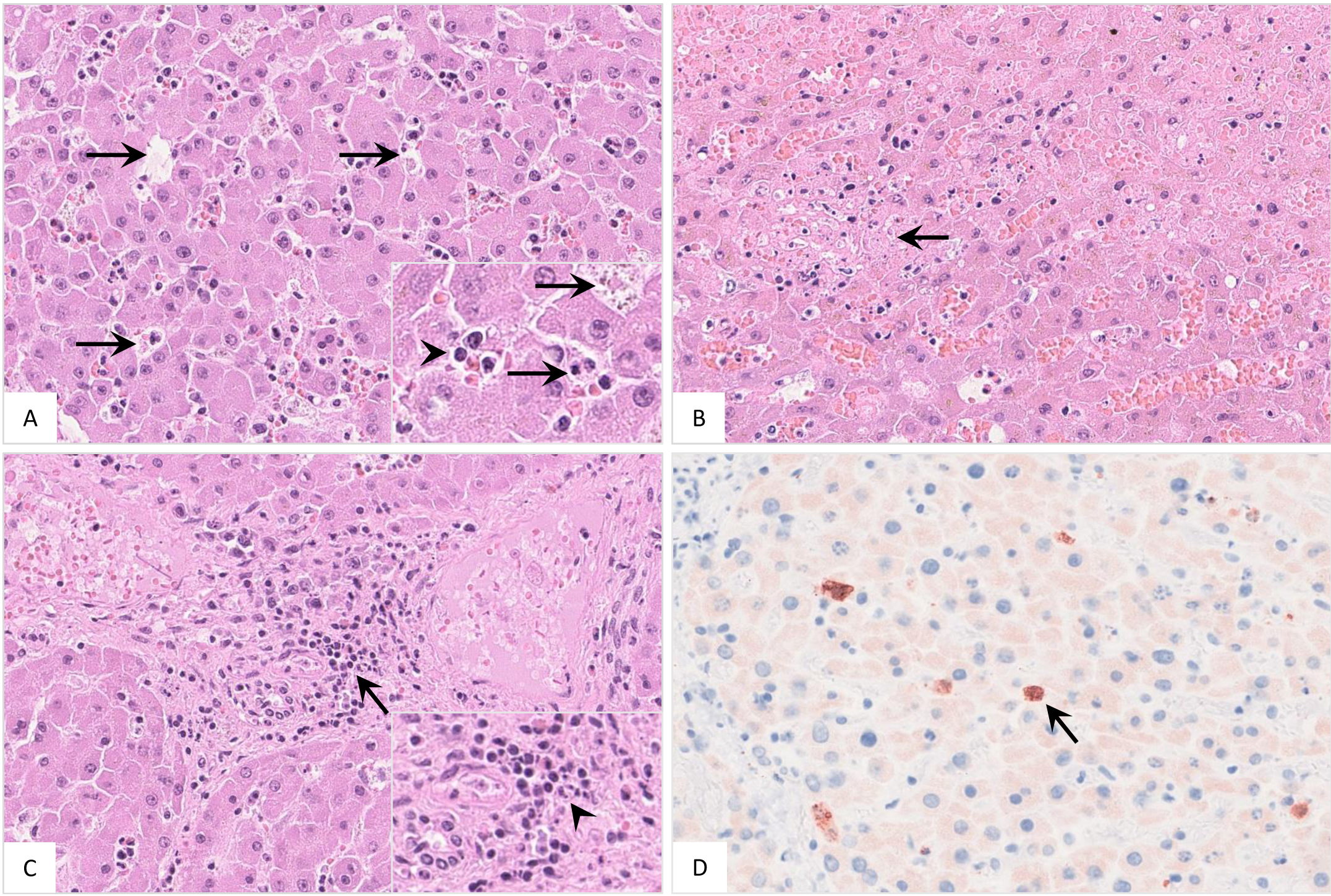

Supplement: Supplementary file 1 [file pathogens-11-01386-s001.zip › Fig S4.tif]

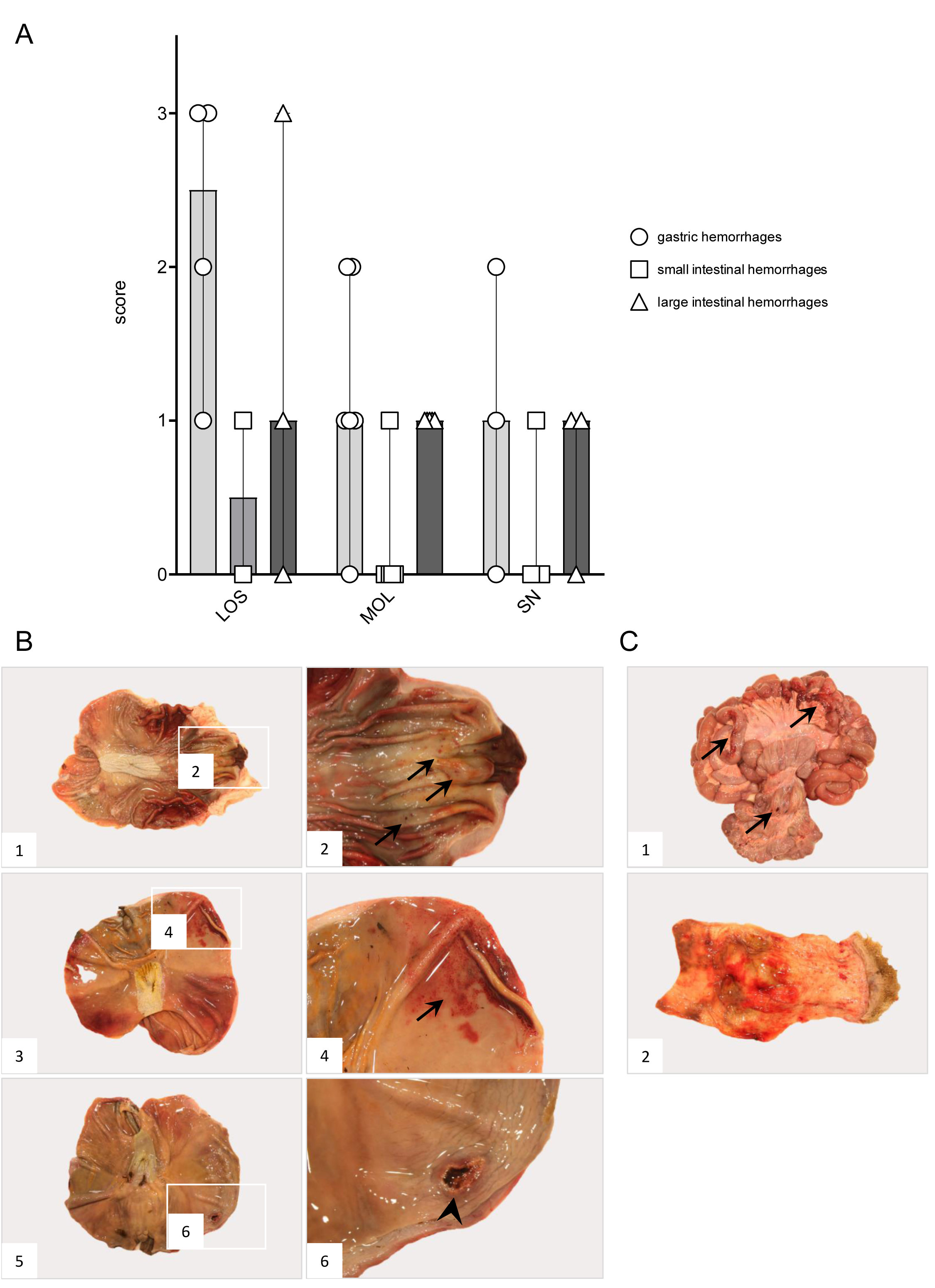

Supplement: Supplementary file 1 [file pathogens-11-01386-s001.zip › Fig S5.tif]

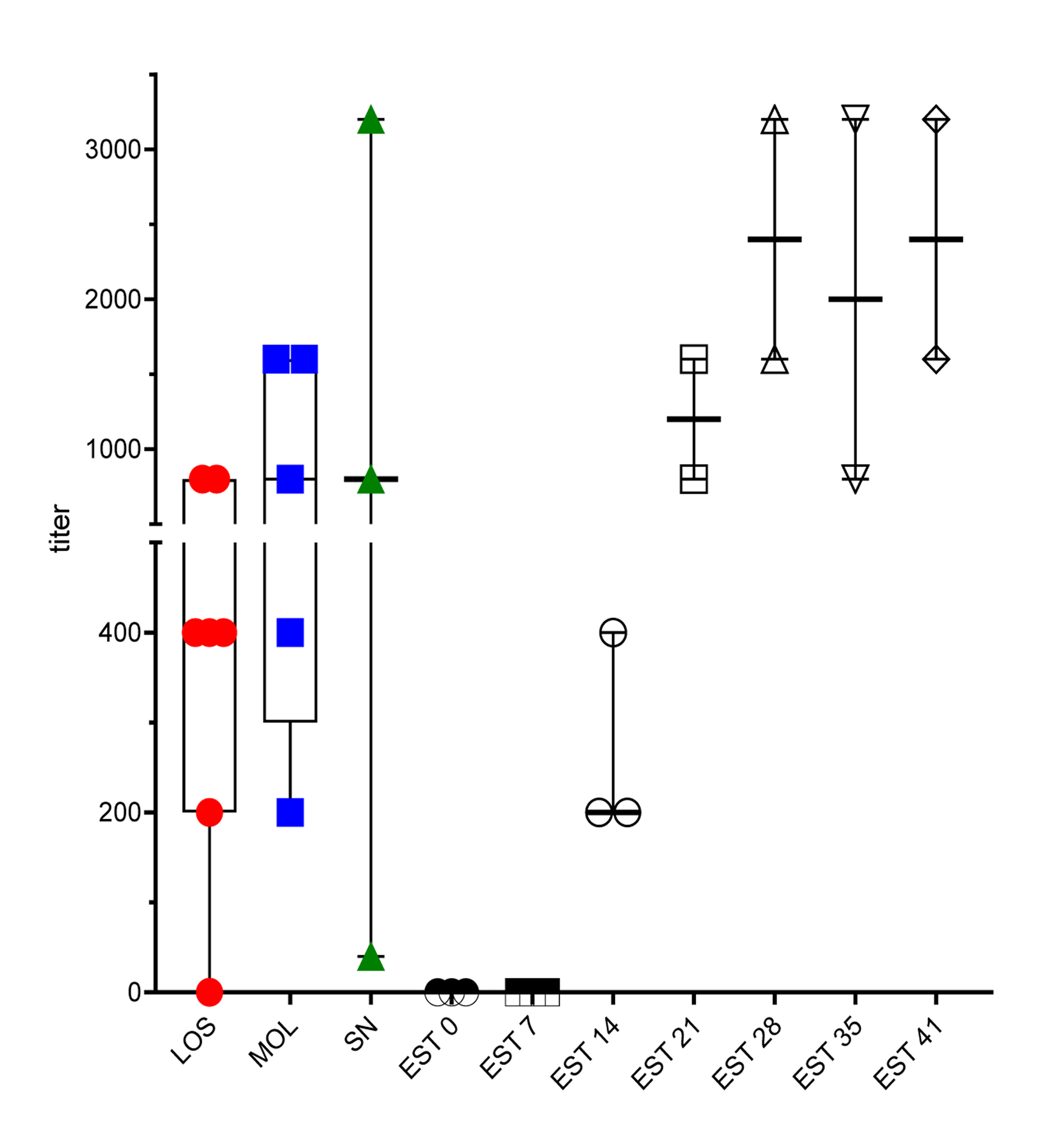

Supplement: Supplementary file 1 [file pathogens-11-01386-s001.zip › Fig S6.tif]
